# Impact of Chelating Agent Choice on Growth Kinetics and Defect Chemistry in Sol–Gel-Synthesized Li- and Mn-Rich Layered Cathodes

**DOI:** 10.1021/acsami.5c19987

**Published:** 2026-03-12

**Authors:** Rabail Badar Abbasi, Marjan Bele, Giuliana Aquilanti, Jasper Rikkert Plaisier, Anton Meden, Luis Miguel Guerrero Mejía, Robert Dominko, Elena Tchernychova

**Affiliations:** a Department of Materials Chemistry, 68913 National Institute of Chemistry, Hajdrihova 19, Ljubljana 1000, Slovenia; b Faculty of Chemistry and Chemical technology, 112794 University of Ljubljana, Ljubljana 1000, Slovenia; c ALISTORE-European Research Institute, Amiens 80039, France; d 18474 Elettra-Sincrotrone Trieste S.C.p.A., s.s. 14 km 163.5, Basovizza, Trieste 34149, Italy

**Keywords:** Li- and Mn-rich layered oxides, oxygen vacancies, stacking faults, chelating agents, redox behavior, cathode design, Li-ion batteries

## Abstract

The electrochemical performance and structural stability of Li- and Mn-rich layered oxide cathodes are critically influenced by synthesis conditions, yet the roles of chelating agents and defect chemistry remains elusive. In this study, we systematically investigate Li_1.2_Mn_0.54_Ni_0.13_Co_0.13_O_2_ cathode powders synthesized via the sol–gel method using citric acid or oxalic acid as the chelating agent, each calcined at 850 and 900 °C. Despite introducing greater initial disorder, oxalic acid-derived samples, particularly the one calcined at 900 °C, demonstrate improved electrochemical stability and capacity retention. *Operando* XRD reveals that this material undergoes a pronounced unit cell expansion during the first cycle, a response linked largely to the mobility and homogenization of oxygen vacancies introduced during the synthesis. This structural flexibility accommodates redox-driven strain during cycling, which limits Li/TM mixing and enables over-reduction of Ni, as confirmed by *operando* XANES and *ex situ* EXAFS. These results highlight that oxygen vacancy mobility during cycling dominates the effects of the initial structural order, where different types of defects are present, and plays a decisive role in governing redox pathways and cycling stability. The selection of the precursor allows tailoring introduction of these defects without postsynthesis treatment. This study provides a comprehensive framework for designing high-performance Li- and Mn-rich layered oxide cathodes by tuning synthesis chemistry to engineer beneficial structural disorder and defect dynamics.

## Graphical abstract

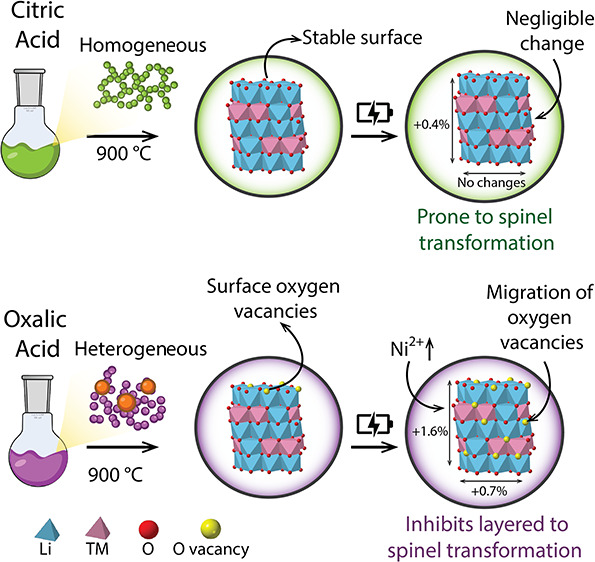

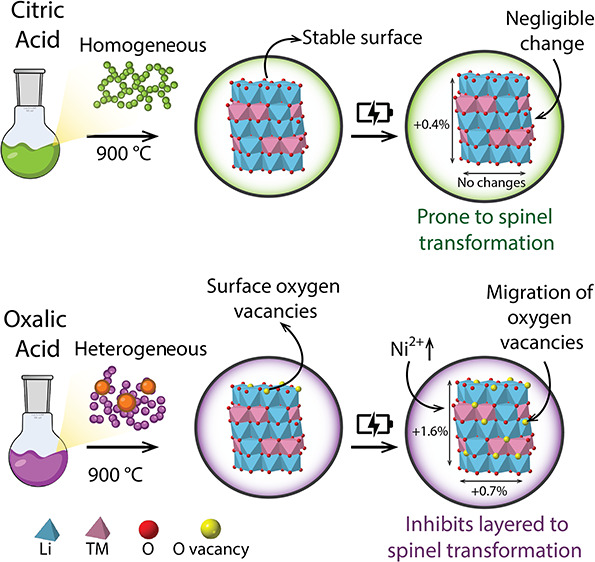

## 1 Introduction

Lithium-ion batteries (LIBs) are the dominant energy storage technology, powering a wide range of applications from portable electronics to electric vehicles.^1,2^ As technology advances, the demand for a higher energy density in LIBs continues to grow. Since cathode materials largely determine the energy density, improving battery performance often relies on developing new chemistries or modifying existing cathodes.^3,4^

Li- and Mn-rich layered oxides (LMR oxides) have attracted attention due to their high specific capacity (>250 mAh/g) and energy density (∼1000 Wh/kg).^5^ The electrochemical behavior of LMR oxides is described by the charge compensation of cationic and anionic (oxygen) redox reactions, which yields complicated reaction kinetics.^6^ Issues such as persistent voltage decay, irreversible oxygen release, and structural instability limit the commercial uses of LMR oxides. Most recent studies correlate voltage decay with the layered to spinel phase transition caused by transition metal (TM) migration.^7–9^ TM migration is linked with structural instability induced by oxygen anionic redox.^10–12^ Therefore, to achieve the best performance from LMR oxides, the understanding and control of anionic redox is of importance. Anionic redox is affected by the various defects present in the structure, among which stacking faults and oxygen vacancies play a significant role.

Stacking faults have been demonstrated to enhance oxygen redox activity, through the alteration of the local environment of oxygen atoms, specifically the elongation of Li–O bond (which also reduces Li^+^ migration barrier), and the decrease of the Li–O–Li bond angle.^13^ With the increase in oxygen activity, the corresponding Mn activity is also increased, and aggressive delithiation (*x* > 1.75 in Li_2–*x*_MnO_3_) can lead to irreversible structural changes.^14^ On the other hand, stacking faults have been shown to hinder Li^+^ diffusion across the TM layers due to a higher energy barrier for out-of-plane diffusion pathways.^15^ Therefore, while stacking faults can enhance oxygen redox, their detrimental effects on Li^+^ diffusion and structural stability emphasize the need to achieve a balance in order to fully exploit their benefits.^15,16^

Oxygen vacancies represent another critical type of defect that can modulate the local electronic structure and induce expansion of the unit cell due to weakened interlayer interactions. Oxygen vacancies have been preferentially introduced into LMR oxides to create a softened structure, which allows for decreased and reversible oxygen anionic redox chemistry.^17–19^ Oxygen vacancies not only increase the distance between planes and hinder TM migration during cycling, they reduce Mn, which then can take part in the charge compensation.^17^ To introduce oxygen vacancies into the structure, postprocessing steps are typically required, making the overall process more time-consuming and less practical.^17,20^

In addition to their functional impact on performance, the origin and evolution of defects such as stacking faults and oxygen vacancies during synthesis are equally critical areas of investigation. Understanding the parameters that govern defect formation, such as precursor chemistry, chelation strength, and thermal conditions, is essential for gaining better control over final material properties. Stacking faults, for instance, are known to be thermodynamically unfavorable and can be reduced through prolonged high-temperature calcination or by carefully selecting precursor chemistry, as shown in prior studies.^13,21–23^ In contrast, oxygen vacancies are often introduced through postsynthetic treatments, such as surface modification or chemical reduction,^20,24,25^ which can complicate the synthesis workflow and reduce scalability. While both types of defects significantly influence anionic redox and structural stability, a comprehensive understanding of how they form in tandem during synthesis is still lacking. To the best of our knowledge, no study has systematically investigated the coevolution of stacking faults and oxygen vacancies during synthesis and their combined influence on electrochemical behavior in LMR oxides.

To address this gap, we employed the sol–gel synthesis method, which offers several advantages for controlling structural evolution at the atomic scale. Compared to solid-state routes, sol–gel synthesis enables molecular-level mixing of precursors, leading to better compositional homogeneity and a lower calcination temperature threshold for phase formation.^26^ Such enhanced control over the early stages of synthesis allows tuning of local environments that may influence defect formation. We further investigated the role of precursor chemistry in defect formation by using two chelating agents of contrasting strength: citric acid and oxalic acid. Citric acid, a stronger and more complexing agent, tends to promote a more homogeneous distribution of metal ions during gelation, while oxalic acid, with its weaker and simpler coordination behavior, induces greater local heterogeneity in the precursor gel. This deliberate contrast allows us to explore how initial compositional disorder influences defect formation and electrochemical behavior in LMR oxides. While previous studies have investigated the role of chelating agents in LMR oxides, they have mainly reported bulk particle features and final electrochemical performance (Table S1). In contrast, our study elucidates the mechanistic pathways connecting precursor chemistry, defect formation, structural evolution during synthesis, and dynamic changes during cycling, highlighting how these factors fundamentally dictate the electrochemical behavior of the LMR oxides. By employing multiscale characterization methods including synchrotron-based X-ray absorption and diffraction techniques, scanning transmission electron microscopy, and X-ray photoelectron spectroscopy, we probe the structural and chemical changes in pristine and cycled samples. While the concentration of stacking faults decreases with increasing temperature for both chelating agents, we find that oxalic acid promotes structural disorder that facilitates the formation of oxygen vacancies during calcination. This indicates distinct and tunable defect formation pathways. During the first electrochemical cycle *operando* XRD measurements, we observe structural expansion, which we link to the migration of oxygen vacancies into the bulk lattice. The unit cell expansion observed upon discharge can facilitate Li^+^ diffusion and suppress TM migration.

## 2 Experimental Section

### 2.1 Materials

Manganese­(II) acetate (Mn­(ac)_2_·4H_2_O, 99.8%), nickel­(II) acetate (Ni­(ac)_2_·4H_2_O, 98%) and cobalt­(II) acetate (Co­(ac)_2_·4H_2_O, 100%), citric acid (HOC­(COOH)­(CH_2_COOH)_2_, anhydrous, 99%), oxalic acid (HO_2_CCO_2_H, anhydrous, 99.6%), ammonia solution (NH_4_OH, 32%), polyvinylidene fluoride (PVDF), *N*-methyl-2-pyrrolidone (NMP, 99%), nitric acid (HNO_3_), and hydrochloric acid (HCl) were provided by Sigma-Aldrich. Lithium acetate (Li­(ac)·2H_2_O, 99%) was purchased from Alfa Aesar. 1 M lithium hexafluorophosphate (LiPF_6_) in ethylene carbonate:dimethyl carbonate:propylene carbonate/EC:DMC:PC (3:1:1) with 1 wt % vinylene carbonate (VC) was used as electrolyte for electrochemical measurements. Carbon-coated lithium titanate (Li_4_Ti_5_O_12_, LTO) was purchased from the NEI Corporation. Carbon black (C65) was used as a conductive additive for the cathode slurries. Celgard 2320 was used as a separator for all cells. Dimethyl carbonate solvent (DMC), supplied by Sigma-Aldrich, was used for washing electrodes and *ex situ* measurements.

### 2.2 Synthesis

We synthesized Li_1.2_Mn_0.54_Ni_0.13_Co_0.13_O_2_ through the sol–gel synthesis method. In this process, stoichiometric amounts of Mn­(ac)_2_, Ni­(ac)_2_, and Co­(ac)_2_ were dissolved in ultrapure water (18.2 MΩ cm^–1^, Milli-Q, Millipore). Separately, a solution of stoichiometric amount of Li­(ac) and chelating agent (citric or oxalic acid) in ultrapure water was prepared, with the molar ratio of chelating agent to metal ion being 1.5 to 1. An excess of 5% of Li­(ac) was added to compensate for Li loss during calcination. This solution was then added dropwise to the TM acetate solution. The pH of the mixture was adjusted to 9 by addition of ammonia solution. The solution was heated to 80 °C, while under stirring, until gelation occurred. The mixture was then dried in a vacuum oven at 200 °C. The powder was then well-ground and calcined first at a temperature of 450 °C for 6 h and then at 850 or 900 °C, for 12 h in air. The heating was done at a rate of 5 °C/min. The sample list is shown below.

**Table 1:** Sample List of LMR Oxides

	temperature (°C)	
chelating agent	850	900
oxalic aid	OA-850	OA-900
ctric acid	CA-850	CA-900

### 2.3 Electrochemical Measurements

Electrochemical measurements were performed using a pouch cell configuration, with either a Li metal or LTO anode, used as the counter electrode. The electrodes were prepared by mixing active material (cathode powder and LTO powder), PVDF, and C65, in the ratio 8:1:1, respectively, dispersed in NMP solution. The cathode active material mass loading on carbon-coated aluminum foil was 2–3 mg/cm^2^, while the LTO anode active material mass loading on copper foil was 4–6 mg/cm^2^ for capacity balancing in the full cell. The cells were assembled in an Ar-filled glovebox. For the galvanostatic tests, the cells were cycled between 2.0 and 4.8 V. The C-rate was determined assuming a theoretical capacity of 250 mAh/g. For cyclic voltammetry measurements, half cells against Li metal were scanned at a scan rate of 0.1 mV/s, unless stated otherwise, in the voltage range of 2.0 to 4.8 V. For galvanostatic intermittent titration technique (GITT) measurements, the cells were preconditioned for 5 cycles at a rate of C/10 to stabilize the cell response. The GITT step consisted of a pulse of current at C/10 for 10 min followed by a relaxation time of 1 h.

The electrochemical measurements were carried out at room temperature (25 °C) by using a potentiostat/galvanostat VMP3 (Bio-Logic, France).

### 2.4 Physicochemical Characterization

Chemical composition of the synthesized LMR oxides was analyzed by using inductively coupled plasma-optical emission spectroscopy (ICP-OES). For sample dilution and preparation of standards, ultrapure water and ultrapure acids (HNO_3_ and HCl) were used. Standards were prepared in-house by dilution of certified, traceable, inductively coupled plasma (ICP)-grade single-element standards (Merck CertiPUR). Before the ICP-OES analysis of bulk ceramics, each sample was weighed (approximately 10 mg) and digested by dissolving it in concentrated HCl (5 mL). Samples were then diluted with 2% vol HNO_3_ until the concentration was within the desired concentration range.

We recorded powder X-ray diffraction (XRD) data using a monochromatic X-ray beam at ∼15 keV (0.8265 Å), over the 2θ range of 8–60° with a step size of 0.005°, at the MCX beamline at Elettra Synchrotron located in Trieste, Italy.^27^ The powder samples were measured in a capillary in a transmission geometry. Rietveld refinement of the XRD data was done using TOPAS-Academic.^28^ For quantification of stacking faults, refinement of structure models with stacking faults, adapted from Serrano-Sevillano et al.,^23^ was carried out using FAULTS software.^29^ For *operando* measurements, the cells were measured in pouch cell configuration in transmission mode using a marCDD detector. The XRD patterns were recorded consecutively at a range of 5° < 2θ < 42°, with an exposure time of 30 s.

The samples for transmission electron microscopy (TEM) were prepared by dispersing the cathode powder in isopropanol alcohol and dropping the dispersion on lacey-carbon-coated copper TEM grids. The samples were examined by a JEM-ARM200CF probe Cs-corrected scanning transmission electron microscope (STEM) equipped with a cold field emission electron source operated at 80 kV. Electron energy loss (EELS) analysis was performed by using a QuantumGIF imaging filter (GATAN, Plesanton, USA). Elemental distribution was assessed by energy-dispersive X-ray spectroscopy in STEM mode (STEM-EDX).

Samples for scanning electron microscopy (SEM) were prepared by depositing dry powder on carbon tape. Imaging was conducted on an FE-SEM Supra 35 VP Carl Zeiss, at an accelerating voltage of 3 kV, with the use of an InLens detector. For the cycled electrode, an accelerating voltage of 1 kV and secondary electron (SE) detector were used for imaging.

X-ray absorption spectroscopy (XAS) measurements were performed in transmission mode at the XAFS beamline at Elettra Synchrotron located in Trieste, Italy.^30^ The white beam was monochromatized using a fixed exit monochromator using a pair of Si (111) crystals. Reference samples consisting of metallic manganese, nickel, and cobalt foil were used for energy calibration in each scan. For pristine powders, pellets were prepared of homogeneously mixed sample and PVDF, of specific weight percentage, according to the element to be measured (Mn, Ni, and Co). For cycled electrodes, the electrodes were disassembled, washed three times with DMC solvent, and dried overnight, after which they were sealed in pouch cells. The samples were sealed in argon to avoid contamination during transportation. The data was processed using the Athena software program.^31^ The extended X-ray absorption fine structure (EXAFS) data were fitted using Artemis software.^31^

X-ray photoelectron spectroscopy (XPS) was performed using a Versaprobe 3 AD (Phi, Chanhassen, US) with a monochromatic Al Kα_1_ X-ray (1486.7 eV) excitation source. The electrodes were placed on nonconductive double tape. High-resolution spectra were measured at a 27 eV pass energy and steps of 0.05 eV on a 200 μm spot size. Charge neutralization was used, and the energy scale of the XPS spectra was corrected by shifting the C 1 s peak of carbon to a binding energy of 284.8 eV. The XPS spectra were analyzed using the Ulvac-PHI Multipak software.

## 3 Results and Discussion

### 3.1 Electrochemical Performance

The synthesized cathode powders were evaluated in half-cell configurations against Li metal to assess their electrochemical performance, as shown in Figure 1. In the first charge cycle (Figure 1a), all of the samples exhibit a characteristic S-shaped voltage profile. The sloping region below 4.5 V corresponds to the cationic redox activity, primarily involving Ni^2+/3+/4+^ and Co^2+/3+^. Above 4.5 V, the plateau region is attributed to anionic (oxygen) redox processes, which can lead to irreversible oxygen release at the end of the first charge.^32^

**Figure 1:**
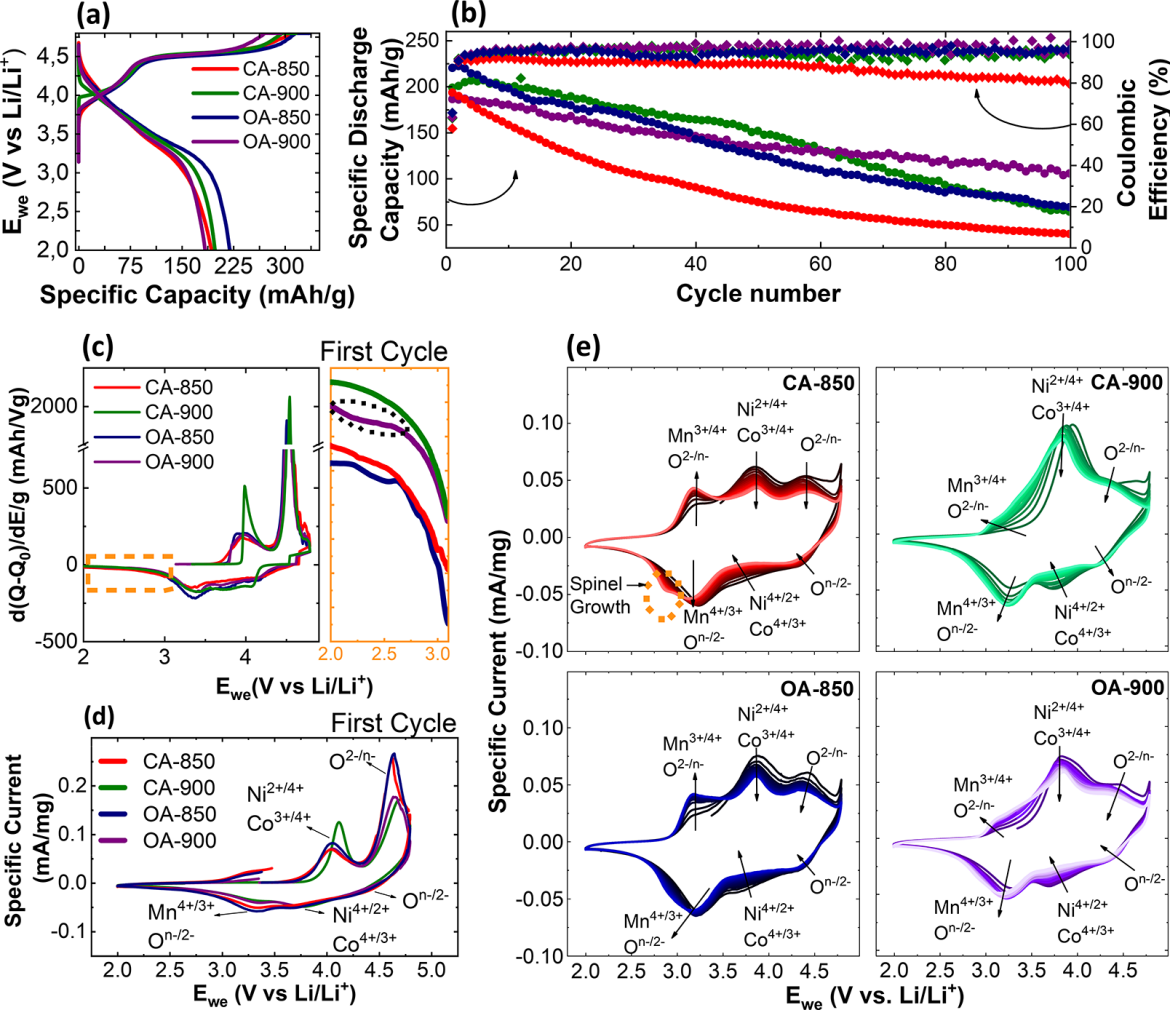
(a) Galvanostatic charge/discharge (GCD) curve at the first cycle, (b) long-term cycling performance and Coulombic efficiency, (c) first cycle differential capacity profiles, (d) first cycle cyclic voltammetry curve of all samples at a scan rate of 0.1 mV/s, and (e) 2nd to 15th cyclic voltammetry curves. All cells were cycled against Li metal in the voltage range 2.0–4.8 V. GCD measurements were carried out at a rate of C/10.

Table 2 summarizes the charge and discharge capacities of the four synthesized samples (see Table 1). The initial charge capacities range from 296 to 337 mAh g^–1^, while discharge capacities span from 183 to 219 mAh g^–1^. Notably, the high-temperature samples (OA-900 and CA-900) exhibit slightly lower charge capacities than their low-temperature counterparts. This trend may be attributed to the reduced amount of electrochemically accessible Li^18^ due to partial densification and/or Li volatilization at elevated synthesis temperatures, proven by ICP-OES measurements (Table S2, Note 1). Upon discharge, all samples exhibit significant capacity loss, primarily due to oxygen redox activity at high voltages, which can induce oxygen release and irreversible structural rearrangements in the cathode.^21^

**Table 2:** Specific Charge/Discharge Capacities and Long-Term Efficiency of LMR Oxides vs Li Metal

sample	initial charge capacity (mAh/g)	initial discharge capacity (mAh/g)	initial capacity loss (%)	100th cycle discharge capacity (mAh/g)	Coulombic efficiency after 100 cycles (%)
OA-850	337	219	35	68.51	95.9
OA-900	296	183	38	105.28	99
CA-850	335	192	43	39.38	79
CA-900	316	198	37	63.64	93.6

To assess long-term electrochemical performance, we cycled the cathodes for 100 cycles at a rate of C/10, with the discharge capacity and Coulombic efficiency shown in Figure 1b. Among all samples, we observe that OA-900 displays the most stable cycling behavior, retaining over 100 mAh g^–1^ after 100 cycles with a Coulombic efficiency close to 99%. In contrast, CA-850 exhibits the poorest retention, dropping below 40 mAh g^–1^. To analyze the long-term stability of the best performing cathodes (OA-900 and CA-900) in a full cell, we cycled them at a faster C-rate against the LTO anode, as seen in Figure S1. The morphology of the OA-900 cathodes after 150 cycles is shown in Figure S2, where the particles retain their original morphology and show no signs of cracking. Overall, we find that cathodes synthesized using oxalic acid demonstrate higher cycling stability compared to those prepared with citric acid.

To further understand the underlying electrochemical processes contributing to the observed performance differences, we examined the differential capacity (d*Q*/d*V*) plots in the first cycle, a portion of which is highlighted in Figure 1c. In the discharge plot, we can see an additional plateau at ∼2.4 V for OA-850 and OA-900; however, it is more pronounced in OA-900. This plateau is related to the reduction/activation of Mn^3+^ in the initial state.^17,33^ The presence of reduced Mn form is related to oxygen vacancies in the structure, which will be discussed further in Section 3.4.

To gain insight into the redox reactions governing the electrochemical behavior, we performed cyclic voltammetry on all four cathodes. In the first cycle, as seen in Figure 1d, we can observe two main redox peaks on oxidation, at ∼ 4.1 and ∼ 4.7 V, which are attributed to TM oxidation (Ni^2+/3+/4+^ and Co^2+/3+^) and anionic oxidation (O^2–^/O^*n*–^), respectively.^6,32,34^ During the corresponding reduction, we can see three peaks at ∼4.5, 3.75, and 3.25 V, which are assigned to reduction of oxygen (O^*n–*/2–^), nickel and cobalt (Ni^4+/3+/2+^ and Co^3+/2+^), and manganese and oxygen (Mn^4+/3+^ and O^*n*–/2–^), respectively. In subsequent cycles for all samples, a new oxidation peak at 3.25 V emerges, which is due to the oxidation of manganese and oxygen (Mn^3+/4+^ and O^2–/*n–*^).^35^

We observe distinct differences in redox behavior across the four samples, with the differences being more evident between temperatures. At lower temperature (OA-850 and CA-850), the anionic oxidation peak is more intense, in comparison to their higher temperature counterparts (OA-900 and CA-900), which relates to the shorter high voltage plateau seen in Figure 1a of the latter. In subsequent cycles (Figure 1e), we see the Mn^3+/4+^ peak grow rapidly at lower temperature samples, which can be associated with the need for charge compensation following more intense anionic redox activity. Since Mn is more redox-active in these samples, it promotes spinel phase formation below 3 V (as seen in Figure 1e), which is known to degrade the cathode over time.^36–38^

The diffusion coefficient of Li^+^ (*D*_Li+_) in the cathode materials was investigated by using GITT measurements, as shown in Figure S3. The equation used to calculate *D*_Li+_ is derived from Fick’s laws of diffusion; further details are provided in Note 2 of the SI. OA-900 and CA-900 exhibit higher *D*_Li+_, compared to their lower temperature counterparts, although the *D*_Li+_ remains on the same order of magnitude across the four samples. During discharge, OA-850 and CA-850 show an increase in *D*_Li+_ at 3 V, correlating with the formation of spinel phase in these samples as seen in Figure 1e. Overall, OA-900 exhibits the highest *D*_Li+_ during both charge and discharge processes, which is consistent with its higher reversible capacity and Coulombic efficiency (Table 2). A detailed analysis of the GITT results is listed in Note 2 in the SI.

To understand the origin of these distinct electrochemical behaviors, we examined how the synthesis process affects the final structure and related these findings to electrochemical performance. We investigated how the choice of chelating agent, citric acid versus oxalic acid, affects the thermal evolution of the precursors during synthesis.

### 3.2 Structural Evolution during Synthesis

The chelating agent not only coordinates with the metal ions but also governs the homogeneity of the initial sol–gel matrix, which directly influences defect formation in the final oxide. A strong chelating agent, such as citric acid, forms a stable and uniform network that ensures a homogeneous distribution of metal precursors. In contrast, a weak agent, such as oxalic acid, results in a less uniform matrix, encouraging the segregation of metal species and formation of intermediate phases.^26^ These differences in the early gel structure ultimately shape the type and distribution of defects, such as oxygen vacancies and stacking faults, in the final LMR oxide.

To investigate the chelating ability of oxalic and citric acid, we characterized the dried gel (xerogel) through XRD as shown in Figure 2a,b. The XRD pattern of the citric acid-derived dried foam (Figure 2a) exhibits a singular broad peak from the borosilicate capillary tube, while the XRD pattern of the oxalic acid-derived foam (Figure 2b) exhibits multiple peaks that are indexed to metallic oxalates and hydroxides. From these observations, it is evident that citric acid, being a flexible triprotic acid, is able to create stable metal-chelate chains and discourages early precipitation of phases, supporting the formation of a uniform organic matrix with numerous evenly dispersed sites for nucleation. In contrast, oxalic acid, a rigid diprotic acid, provides weaker chelation, allowing the early precipitation of oxalates and hydroxides during gel formation, creating heterogeneity in the starting matrix.

**Figure 2:**
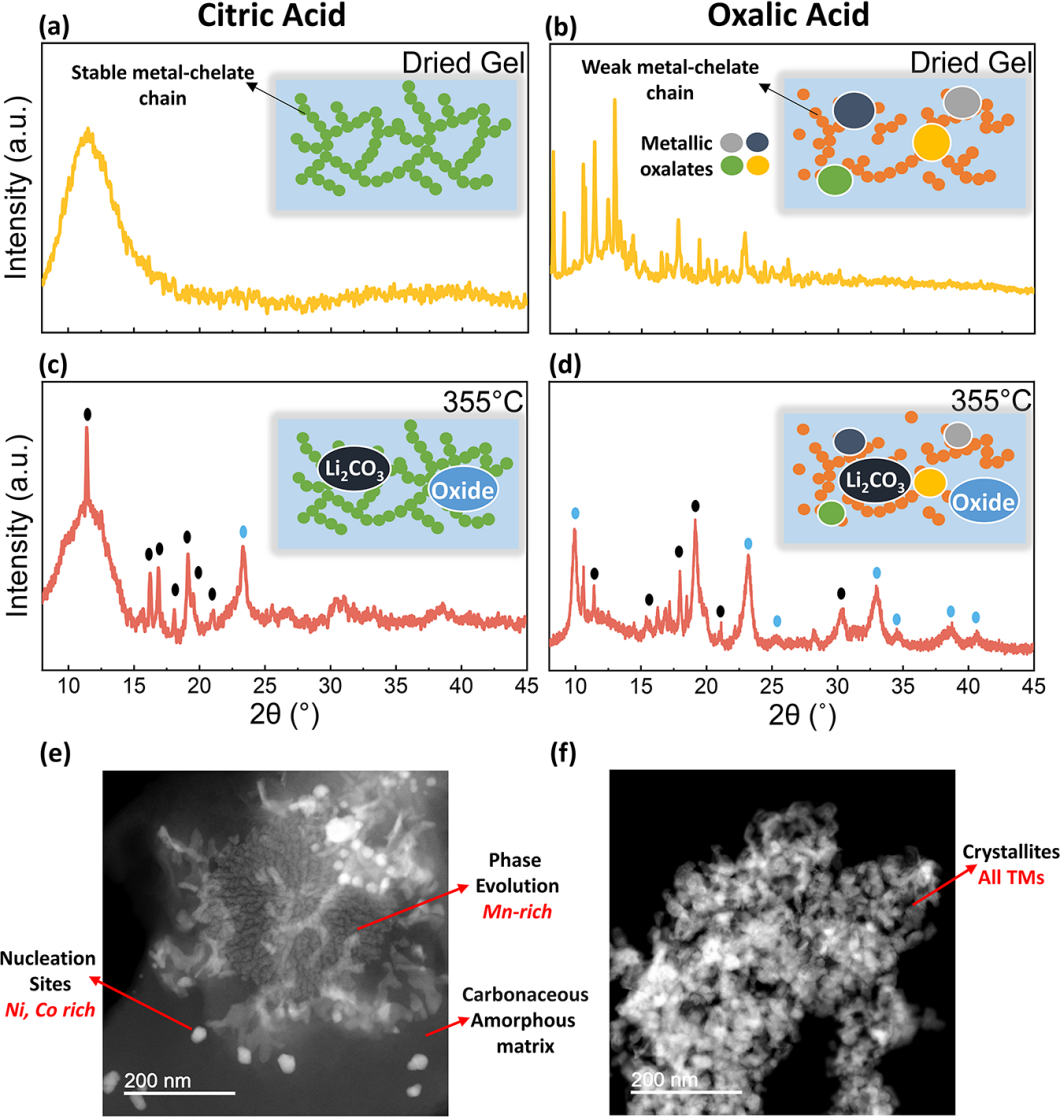
XRD patterns during synthesis of LMR oxides using (a,c) citric acid and (b,d) oxalic acid as the chelating agent at different stages of heating with corresponding illustrations showing the presence of different phases. HAADF-STEM image of (e) citric acid and (f) oxalic acid systems, at 355 °C.

As the gels are heated up to 355 °C, the combustion of the organic matrix is initiated, which decomposes into CO_2_ or CO.^39^ For citric acid (Figure 2c), a few peaks emerge in the XRD pattern, which are largely indexed to Li_2_CO_3_, which is formed in the following reaction.^40^

$$2\mathrm{LiOH}+{\mathrm{CO}}_{2}\to {\mathrm{Li}}_{2}{\mathrm{CO}}_{3}+H_{2}O$$

The peak at 22.7° is indexed to a layered oxide structure, indicating the start of the formation for a metallic oxide. Moreover, the persistent amorphous background suggests the continued presence of the organic matrix, which promotes the controlled phase evolution. In contrast, for the oxalic acid-derived foam at 355 °C, several oxalate-related peaks persist, and new phases such as Li_2_CO_3_ and various metallic oxides begin to emerge. The higher intensity and number of peaks for the metallic oxide phase indicate a faster reaction progression compared with the citric acid system.

To directly observe the reaction evolution, we imaged both samples at 355 °C using high-angle annular dark-field–STEM (HAADF-STEM). In the citric acid-derived sample (Figure 2e), an amorphous matrix is observed, within which various embedded structures are present. To determine their composition, we performed STEM-EDX mapping (Figure S4). The analysis shows that the surrounding matrix is primarily carbon-rich, while the embedded structures exhibit segregation of Ni, Co, and Mn. Specifically, the spherical features are enriched in Ni and Co, whereas the ‘flower-like’ structures are mainly composed of Mn and O. This observation aligns with the lower activation energy for the decomposition of Mn acetates, making Mn the first element to oxidize during thermal treatment.^41,42^ In contrast, the oxalic acid-derived sample exhibits numerous small crystallites with little to no amorphous matrix. STEM-EDX mapping of this sample shows a more random distribution of all three TMs, supporting a heterogeneous synthesis pathway, consistent with the XRD observations.

In addition to XRD, changes in the local structure were monitored by using XAS, as shown in Figure S5. For the citric acid-derived samples, the Fourier transform (FT) moduli of the EXAFS data at 200 °C display features typical of amorphous systems, confirming the XRD observations. At 355 °C, an increase in the crystalline fraction is observed, along with a reorganization of the local order around the transition metals, particularly around Co and Ni. In the case of the oxalic acid system, a certain degree of crystallinity is already present at 200 °C, which is also supported by XRD data. As the temperature increases, the local structure becomes more ordered and the crystallinity improves, as evidenced by the emergence of a prominent second-shell peak in the FT moduli. The presence of multiple intermediate phases suggests a more complex (or disordered) reaction pathway due to the absence of an initially homogeneous organic matrix. This would result in more heterogeneous nucleation, which tends to be faster and less controlled than homogeneous nucleation.^43^

### 3.3 Local and Bulk Chemistry and Structural Order

The analysis of the final synthesized samples was done through XRD, HAADF-STEM, and STEM-EELS. As shown in the XRD pattern in Figure 3a,b, the main Bragg reflections for all samples correspond to a layered rhombohedral phase (*R*3̅*m*), while the broad and asymmetric diffraction peaks at 2θ values of 11.2–14.5° indicate honeycomb Li/TM ordering (monoclinic phase (*C*2/*m*)) within the TM layer. The asymmetric shape of these peaks is due to the presence of stacking faults in the structure,^44–46^ which are depicted in Figure 3c,d. Stacking faults are deviations from the ideal stacking symmetry of atomic planes, often due to lateral shifts in the Li-TM layers. These shifts occur as a mechanism to relieve local strain or accommodate structural distortions, especially in materials with high cation disorder or nonstoichiometry, as in Li-rich oxides.^47^ With the increase in stacking fault density, there is a gradual decrease in the intensity and an increase in broadening of the peaks in the low angular region of the XRD pattern.^48^ The stacking fault concentration, determined by structural refinement using FAULTS (refinement model details in Note 3 in the SI), as seen in Figure S6, and the refined parameters listed in Tables S3–S7, establishes that increasing calcination temperature leads to a reduction in stacking fault concentration for both chelating agents. This behavior is consistent with increasing crystallization at higher temperatures. Stacking faults were also seen in STEM images for all samples (Figure S7). In the regions where a stacking fault is present, the distance between the metallic layers is shortened, as seen in the labeled interplanar distances in Figure 3c. This occurs as the M–O (metal–oxygen) octahedra are distorted due to the disruption in the stacking sequence. To obtain detailed structural parameters, we carried out Rietveld refinement using a *R*3̅*m* space group, for simplicity. The detailed refinement data are listed in the SI (Note 4, Table S8, Figure S8) and Table 3. The lattice parameters indicate a trend that the *c* parameter increases with temperature for both samples, consistent with a decrease of stacking fault concentration.

**Figure 3:**
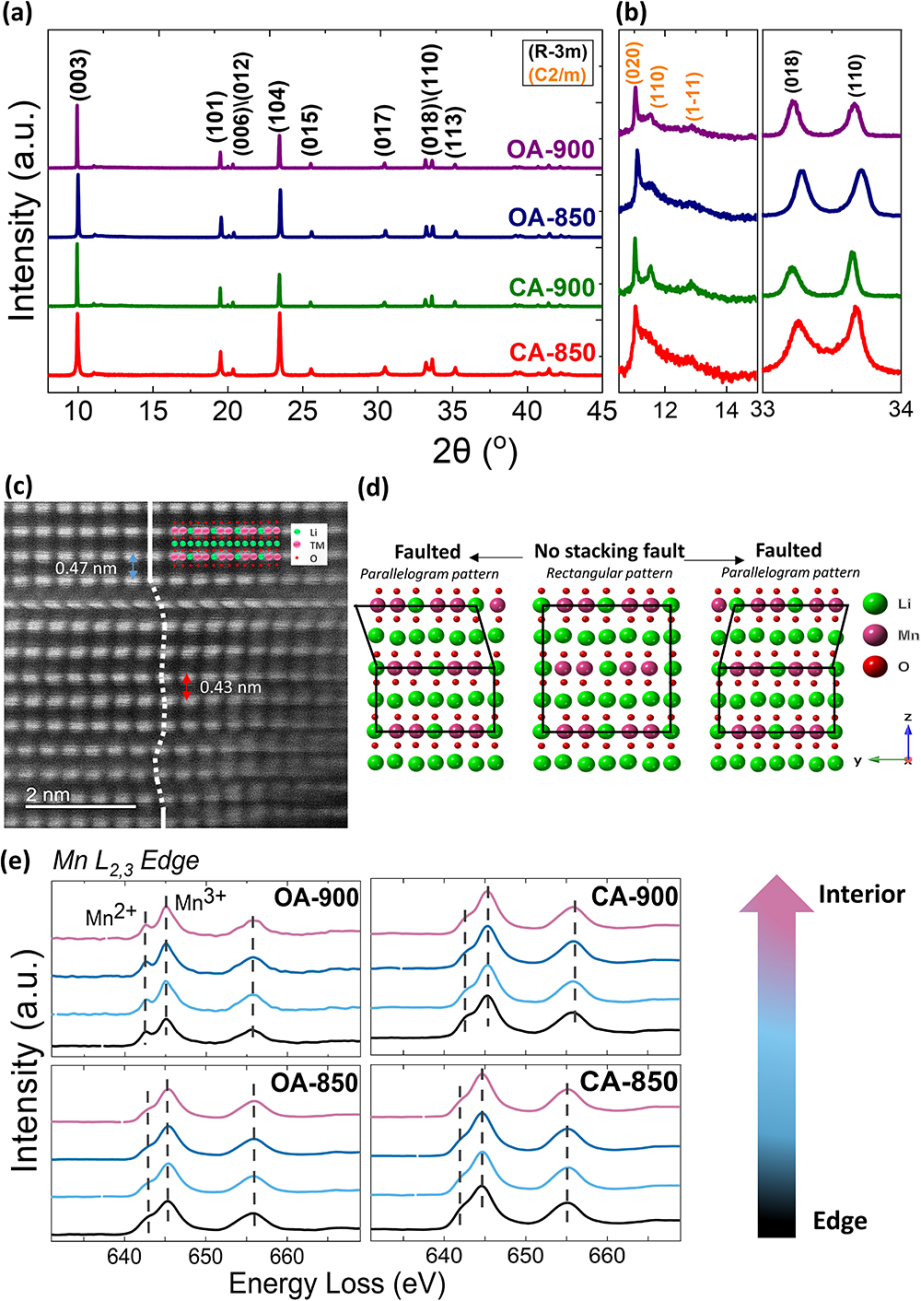
(a) XRD patterns of final calcined LMR oxide samples and corresponding (b) enlarged sections. (c) HAADF-STEM image of the OA-900 sample at (100) zone axis showing stacking faults and changes in interplanar spacing. (d) Ideal monoclinic structure of LMR oxide with a rectangular pattern and the corresponding parallelogram pattern arising due to stacking faults. (e) STEM-EELS spectra at the Mn L_2,3_ edge for all the samples, showing the higher intensity of Mn^2+^ signal due to the presence of oxygen vacancies in the OA-900 sample. The STEM-EELS spectra are collected at increments of 5 nm for a total depth of 20 nm.

**Table 3:** Lattice Parameters Calculated by Rietveld Refinement Using the *R*3̅*m* Space Group

sample	*a* (Å)	*c* (Å)	*I*_(003)_/*I*_(104)_	Li^+^/Ni^2+^ intermixing
OA-850	2.852251(45)	14.227998(328)	1.31	0.94(20)
OA-900	2.851357(28)	14.232455(171)	1.92	0.30(16)
CA-850	2.851010(60)	14.208020(500)	1.02	1.55(18)
CA-900	2.851815(27)	14.234097(181)	1.94	0.29(17)

Furthermore, the intensity ratio of (003)/(104) reflections increases with synthesis temperature, indicating a reduction in Li^+^/Ni^2+^ cation mixing.^49^ In layered structures, a higher (003)/(104) ratio reflects improved cation ordering, which is critical for Li^+^ mobility and structural stability. Li^+^/Ni^2+^ intermixing depends on chemical composition and synthesis parameters, such as the choice of chelating agent and calcination temperature.^50,51^ Among the 850 °C samples, the citric acid-derived sample (CA-850) exhibits a notably lower (003)/(104) ratio, suggesting a higher degree of cation disorder, which was calculated through Rietveld refinement (Table 3). This sample also exhibits the highest density of stacking faults (Figure S6). These observations can be directly correlated to the synthesis pathway: citric acid acts as a strong, multidentate chelating agent, forming a homogeneous and amorphous gel that delays crystallization during the early stages of heating. As evidenced by the XRD of the dried foam and intermediate at 355 °C in Figure 2, the persistent amorphous nature and lack of intermediate crystalline phases in the citric acid system imply slower reaction kinetics. This contributes to delayed nucleation and growth of the final oxide phase and the increase in optimal temperature for reduced Li^+^/Ni^2+^ intermixing compared to the oxalic acid-derived samples. At 900 °C, CA-900 and OA-900 exhibit both a higher (003)/(104) intensity ratio and reduced asymmetry in the low-angle XRD region, clear indicators of lower Li^+^/Ni^2+^ cation mixing and reduced stacking fault density, respectively, due to higher temperature allowing for crystallization and ordering. It is important to note that CA-900 exhibits the lowest stacking fault concentration; homogeneous phase evolution favors higher crystallinity.^43^

Another notable feature is the relative intensities of the (018) and (110) diffraction peaks, as seen in Figure 3b. In CA-derived samples, the (110) peak appears more intense, whereas in OA-derived samples, the (018) and (110) peaks are of similar intensities. In layered cathode materials, the (110) plane is especially advantageous, as it runs along the *a*- or *b*-axis and provides an open channel for Li-ion transport and charge transfer.^52,53^ The enhanced intensity of the (110) peak in CA-derived samples suggests a preferred orientation along this plane, implying that more particles are exposed to the (110) facets. Being a low-index plane, the (110) orientation is generally associated with improved redox kinetics and enhanced interfacial stability during both synthesis and electrochemical cycling.

At elevated synthesis temperatures, oxygen can volatilize from the structure, forming oxygen vacancies, particularly at exposed surface planes where oxygen release is more favorable.^54^ Oxygen loss is also dependent on particle size, as a smaller particle size provides a larger surface area for oxygen loss, and vice versa. To examine this, particle sizes were estimated from SEM images (Figure S9). The oxalic acid-derived samples exhibit smaller particle sizes compared with the citric acid-derived samples at both 850 and 900 °C, suggesting a higher degree of surface reactivity and oxygen loss in the oxalic acid systems. A useful indirect method to probe surface oxygen vacancies is STEM-EELS at the TM edges. The formation of oxygen vacancies typically leads to a reduction in the oxidation state of the TM to maintain charge neutrality, which can explain the features related to Mn^3+^ reduction at discharge for OA-900 in the dQ/dV curve in Figure 1c.

STEM-EELS measurements were conducted at the O K edge and the Mn, Ni, and Co L_2,3_ edges for all samples. As the compositions are Mn-rich, the Mn L_2,3_ signal is the strongest and most reliable, as shown in Figure 3e while the rest can be found in Figure S10. At the lower-energy L_3_ edge, a characteristic shoulder is observed, which serves as a fingerprint for Mn^2+^ species.^55^ All samples display this shoulder to varying extents, with OA-900 exhibiting the most pronounced feature, indicating the highest concentration of Mn^2+^ and, by extension, the greatest degree of oxygen vacancies. Furthermore, the shift to lower energy also points toward a higher proportion of Mn^3+^, consistent with increased oxygen deficiency.^56^ This correlates with the earlier onset of crystallization observed in OA-derived samples, which allows more time for oxygen loss from the lattice compared to CA-derived samples, in addition to the exposure of (110) planes in CA-derived samples, which are more stable and would discourage loss of oxygen.

### 3.4 Operando structural and redox evolution

To reveal the changes in the bulk lattice parameters of the four cathode materials, *operando* XRD was performed in a half-cell configuration cycled at a rate of C/10. Figure 4a–d shows the evolution of the (003) and (101) peaks for all samples, which can be used to reflect variations in the *c* and *a* lattice parameter, respectively. The asterisk symbol corresponds to signals from the aluminum in the pouch cell. *D*-spacing is calculated for the two peaks using Bragg's law shown in Figure S11.

**Figure 4:**
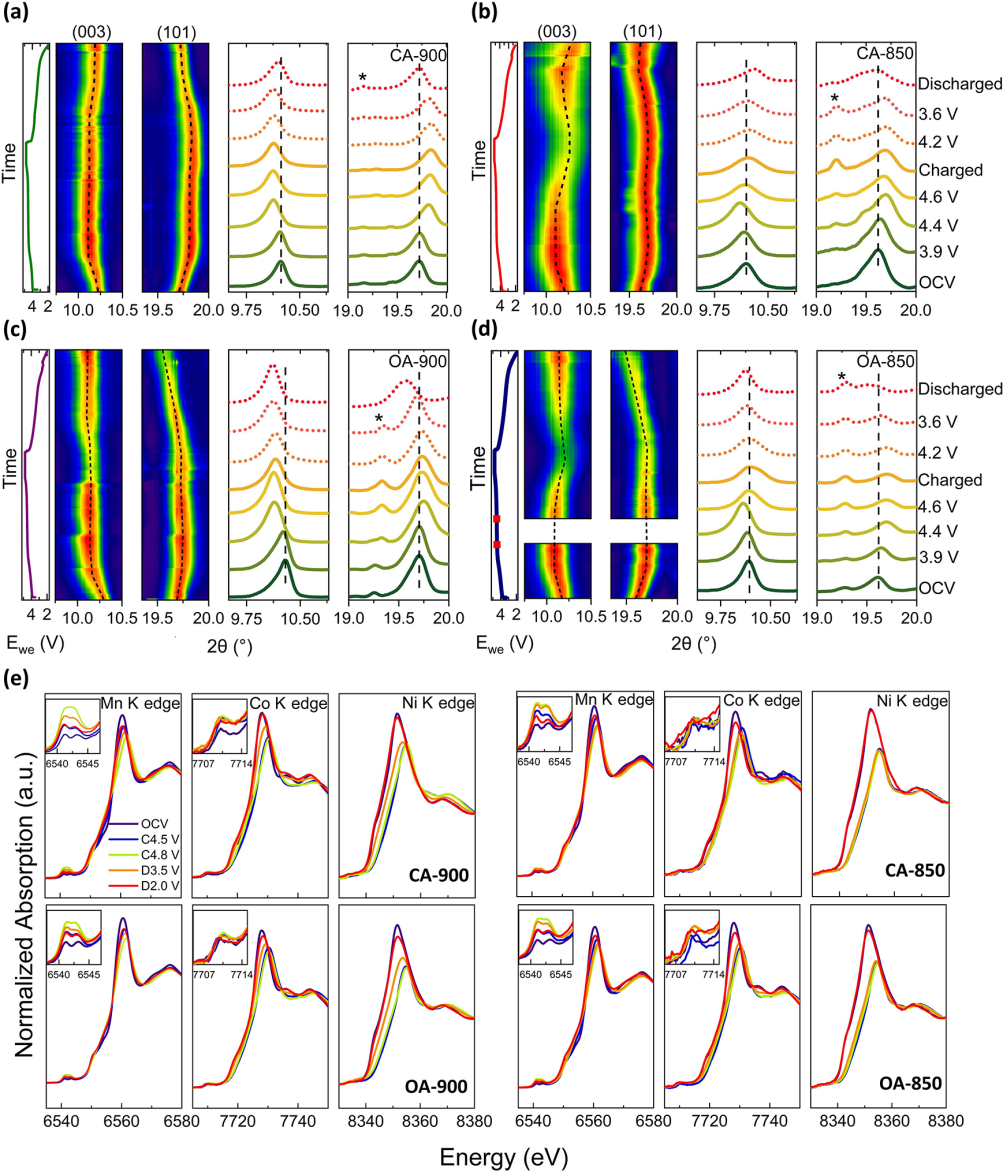
First charge–discharge curve of LMR oxides, with corresponding contour maps and spectra of *operando* X-ray diffraction (XRD) data of (003) and (101) peaks, illustrating changes in lattice parameters, *c* and *a*, respectively, for (a) CA-900, (b) CA-850, (c) OA-900, and (d) OA-850. The asterisk symbol (*) represents the signal from Al in the pouch cell. (e) *Operando* XANES at Ni, Co, and Mn K edge during first charge and discharge for CA-900, CA-850, OA-900, and OA-850, as labeled. All *operando* electrochemical measurements were carried out in half-cell configuration against Li metal, cycled at a rate of C/10 in the voltage range 2.0 to 4.8 V.

Cationic activity can be correlated with changes in the *a* parameter, as it is sensitive to changes in the TM–O bond length in the TM slab/layer.^57^ It is to be noted that the (101) plane is also dependent on the *c* parameter; however, the shifts seen in the (101) peak position are opposite to those seen by the (003) peak position, which is entirely dependent on variations in the *c* parameter. As such, the changes in the (101) plane are mostly dependent on the *a* parameter, and we can correlate these changes to cationic behavior. As the cathode is charged from OCV to 4.5 V, the (101) peak shifts to higher angles, corresponding to contraction of the *a* parameter due to the oxidation of TMs. Above 4.5 V, this peak stabilizes, suggesting minimal further TM oxidation. Upon discharge, as TMs reduce, the TM–O elongate and we see concurrent expansion in the *a* parameter.^58^ Notably, for OA-900, the (101) peak shifts below its initial/pristine position, indicating an overall expansion of the *a* parameter relative to the pristine state (∼0.7% increase in the (101) plane *d*-spacing).

The changes exhibited by the *c* parameter are due to the changes in layer repulsion between (Li,TM)­O_2_ slabs. During delithiation, Li^+^ removal increases electrostatic repulsion, causing the (003) peak to shift to lower angles (*c* parameter expansion). Above 4.5 V, divergent behaviors emerge: while OA-900 and CA-900 show little to no further change, OA-850 and CA-850 exhibit a contraction indicative of structural rearrangement. The difference in behavior above 4.5 V between higher and lower temperature cathodes is due to the higher degree of stacking faults in the latter samples. Prior studies suggest that stacking faults promote oxygen activity and allow deeper delithiation, potentially forming peroxo-like O–O species.^14^ This can lead to irreversible oxygen loss and increased Mn oxidation, consistent with the enhanced Mn^3+/4+^ redox signature seen in CVs (Figure 1d).^13,14^ Upon discharge, all samples show partial recovery of the (003) peak toward the pristine position. However, OA-900 displays a larger *c* parameter at the end of discharge (∼1.6% increase in (003) plane *d*-spacing), potentially due to irreversible structural changes linked to oxygen vacancy migration.^59^ Such increases in unit-cell volume have been attributed to the mobility and redistribution of oxygen vacancies, especially in oxygen-deficient systems such as OA-900. The bulk probing nature of XRD supports this interpretation.

To investigate redox behavior at the elemental level, *operando* XANES was carried out at the Mn, Ni, and Co K edges (Figure 4e). During charging, the Ni and Co edges shift to higher energies, indicating oxidation, while a reverse shift is observed on discharge. In contrast, the Mn K edge position remains relatively unchanged, confirming its minimal role in direct redox. However, noticeable changes in the Mn pre-edge features point to a gradual evolution of local distortion around the Mn. The Mn pre-edge consists of two peaks at ∼6541.0 eV (t_2g_) and ∼6543.0 eV (e_g_), attributed to crystal field splitting in the octahedral environment.^60^ As charging proceeds, the intensity of these peaks increases and their separation narrows; although the shape partially recovers on discharge, the final intensity remains elevated, suggesting irreversible distortion of MnO_6_ octahedra (Figure S12). Interestingly, for CA-850, the intensity of the peaks increases at 4.5 V and does not move significantly at full charge. This suggests an earlier onset of distortion in the MnO_6_ octahedra.

The Co K edge pre-edge, arising from a 1s → 3d electric quadrupole transition, is typically symmetry-forbidden in an ideal octahedral environment but becomes allowed under local distortion.^61^ An increase in pre-edge intensity thus reflects a deviation from the centrosymmetry around Co. In the case of OA-900, the Co pre-edge remains largely unchanged throughout the cycle, indicating reduced distortion and a more stable local structure. Interestingly, the onset of Ni and Co reduction during discharge occurs at higher voltages (∼3.5 V) in the OA-900 and CA-900 samples, compared to their lower temperature sample counterparts, i.e., OA-850 and CA-850. This earlier reduction may be attributed to improved Li^+^ diffusion pathways in high-temperature samples, consistent with reduced stacking fault concentrations.^15^

While *operando* XRD and XANES reveal bulk lattice changes and oxidation state trends, the notable expansion in both *a* and *c* parameters for OA-900 at the end of discharge raises the possibility of over-reduction of TMs. To probe this hypothesis and gain insight into local atomic environments beyond average structural changes, EXAFS analysis was performed on pristine and cycled samples.

### 3.5. *Ex Situ* Characterization of Cycled Electrodes

Figure S13 presents the average bond lengths derived from EXAFS fitting at the Mn, Ni, and Co K edges for three states: pristine, charged, and discharged. The first and second coordination shells correspond to TM–oxygen (TM–O) and TM–TM interactions, respectively. Data were fitted using established models reported in the literature (Note 5 in SI).^62^ The fitting parameters are listed in Table S9. Overall, bond lengths contract during charging and expand upon discharge, in line with the changes in ionic radii that accompany TM oxidation and reduction. Among the samples, OA-900 exhibits notably minimal change in the Ni–TM bond length during charge, suggesting a more stable local structure as Li is extracted.

The relative changes in bond length between the pristine and discharged states are summarized in Figure 5a. OA-900 shows the largest overall expansion in bond lengths upon discharge, which is consistent with the unit cell expansion observed in *operando* XRD. An exception to this trend is seen in the Mn–TM bond, where OA-850 and CA-850 exhibit the greatest increase, which can be related to the oxygen redox mechanism in these samples. As previously discussed (Section 3.3), the lower temperature samples have a higher degree of stacking faults retained in the structure. These defects promote stronger and more irreversible anionic redox activity,^13,14^ which triggers greater Mn redox participation, evidenced by the higher growth of the Mn^2+/4+^ peak pair in the CV data (Figure 1e), for OA-850 and CA-850.

**Figure 5:**
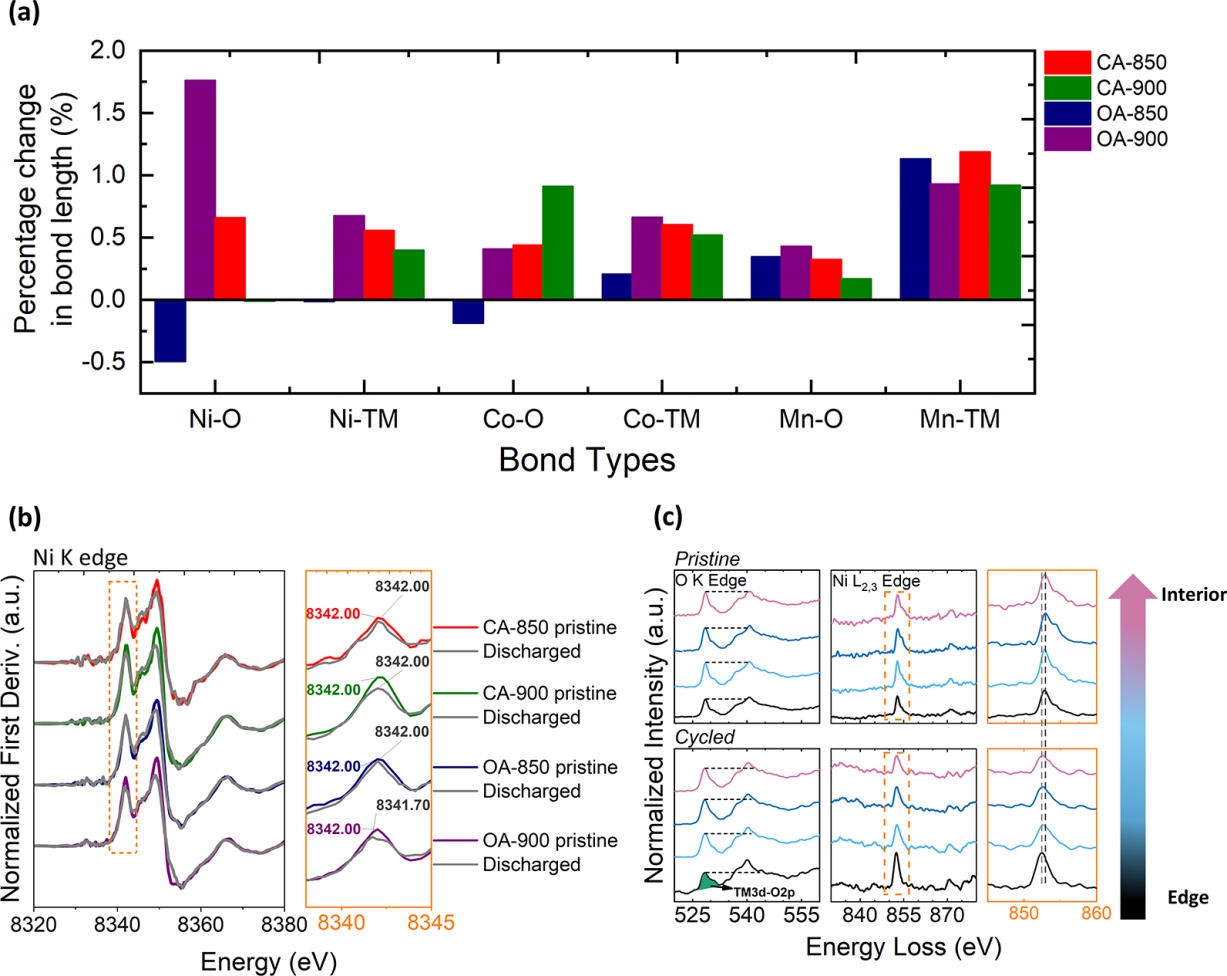
(a) Relative changes in bond length between the pristine and discharged samples, (b) normalized absorption derivative of Ni K edge data for pristine versus discharged states of all samples, and (c) STEM-EELS spectra at O K edge and Ni L_2,3_ edge for OA-900 at the pristine and cycled/discharged state. The STEM-EELS spectra are collected at increments of 5 nm for a total depth of 20 nm.

Among all coordination shells, the Ni–O bond in OA-900 shows the most significant expansion on discharge (1.75% increase). To investigate this further, we examined the valence state of Ni, Mn, and Co at the end of discharge using the first derivative of normalized TM K-edge XANES spectra, as shown in Figure 5b for Ni and in Figure S14 for Mn and Co. The derivative peak indicates the onset of absorption and is associated with the oxidation state. For OA-900, a clear shift of the Ni derivative peak to lower energy is observed in the discharged state, confirming reduction of the average Ni oxidation state compared to the pristine state. In pristine LMR oxides, Ni can be present as both Ni^2+^ and Ni^3+^. While most studies on mixed valence behavior of Ni have focused on LiNiO_2_ systems,^63–65^ similar mixed valency has also been reported in LMR oxides.^66^ This background supports the interpretation of the Ni K-edge shift, where a move to a lower energy in the discharged state likely reflects a higher fraction of Ni^2+^, thereby reducing the average valence state of Ni. This reduction is further supported by the O K edge and Ni L_2,3_ edge STEM-EELS spectra shown in Figure 5c, with the corresponding data for Mn and Co presented in Figure S15a. The prepeak intensity at the O K edge, relative to the main peak, decreases after cycling, as shown in Figure 5c, with the corresponding evolution of the intensity ratio of the main peak to the prepeak shown in Figure S15b; details of the ratio calculation are provided in Note 6 of the SI. The prepeak emerges due to the TM 3d–O 2p hybridization and its reduction in intensity can be attributed to two main, often correlated, reasons: (1) the creation of oxygen vacancies and (2) a reduction in the oxidation state of the surrounding TM.^67^ Consistent with the latter, the Ni L_2,3_ edge in the discharged state shifts up to 0.5 e V lower energy relative to the pristine state, in agreement with the XANES findings. To complement these results, XPS measurements were performed at pristine, charged, and discharged states, after the first cycle. Figure S16 displays the Ni 2p spectra, where the main 2p_3/2_ peak at ∼855 eV contains signals from Ni^2+^ and Ni^3+^ and a satellite peak at ∼862 eV. These features are characteristic of Ni containing LMR oxides.^66^ While deconvolution of the Ni 2p XPS spectrum into Ni^2+^ and Ni^3+^ can lead to ambiguous and nonunique results, the intensity of the satellite peak can serve as a qualitative indicator of Ni^2+^ content, as the two are directly related.^68,69^ From Figure S16, it is evident that after the first discharge of the OA-900 sample, there is a marked increase in the intensity of the satellite peak (more Ni^2+^ content), which confirms an overall reduction in the Ni oxidation state.

The higher fraction of Ni^2+^ at discharge or over-reduction of Ni increases the average ionic radius of Ni and contributes to the observed expansion in both local and long-range structures. Given that Ni exhibits the most substantial change in radius during redox transitions, its over-reduction provides a plausible explanation for the unit cell expansion seen in XRD.

The structural and chemical changes observed after the first cycle arise from the interplay between stacking faults and oxygen vacancies in the pristine OA-900 sample. The lower concentration of stacking faults suppressed irreversible oxygen loss by reducing local distortions in the oxygen sublattice. This is evidenced by the minimal changes observed in the oxygen redox voltage range during *operando* XRD (Figure 4, Figure S11). Oxygen vacancies are known to migrate to the bulk of the particle during cycling,^59^ where they can cause the reduction of Ni species and increase Ni^2+^ content relative to the pristine state.^70^ Due to the heterogeneity in the oxalic acid-assisted system, more oxygen vacancies are formed in OA-900, the migration of which enabled the expansion of the unit cell upon cycling, while the lower degree of stacking faults prevented irreversible oxygen redox,^14^ therefore limiting structural disorder. The resulting enlarged unit cell may inhibit the layered to spinel phase transition, resulting in improved structural stability. In contrast, while CA-900 also promotes reversible oxygen redox, it is prone to Li/TM mixing with prolonged cycling.

To summarize the findings in this study, Figure 6 encapsulates the key differences in defect evolution observed at 900 °C between citric and oxalic acid-based sol–gel-synthesized LMR oxides. The distinct chelating properties of citric and oxalic acid lead to fundamentally different phase evolution pathways: citric acid results in a more homogeneous distribution and slower phase development, while oxalic acid drives a faster, more heterogeneous, and disordered phase evolution. At 900 °C, both systems exhibit a reduction in stacking fault concentration. In the citric acid system, the particles are larger, resulting in lower exposed surface area, and have a preferred orientation along the (110) plane, both of which contribute to a more stable surface. In contrast, the oxalic acid system produces smaller particles, leading to a higher exposed surface area, making the oxalic acid-derived samples more prone to oxygen loss from the surface, creating oxygen vacancies. During the first discharge, the oxygen vacancies become mobile and migrate into the bulk, causing structural expansion and over-reduction of Ni. This increased structural flexibility facilitates Li deintercalation and effectively suppresses the detrimental layered-to-spinel phase transformation by limiting Li/TM intermixing through enlarged interlayer spacing. Overall, these results highlight the crucial role of chelating agent choice in tuning defect structures and phase evolution kinetics, which in turn governs the electrochemical stability and performance of LMR oxides synthesized at high temperatures.

**Figure 6:**
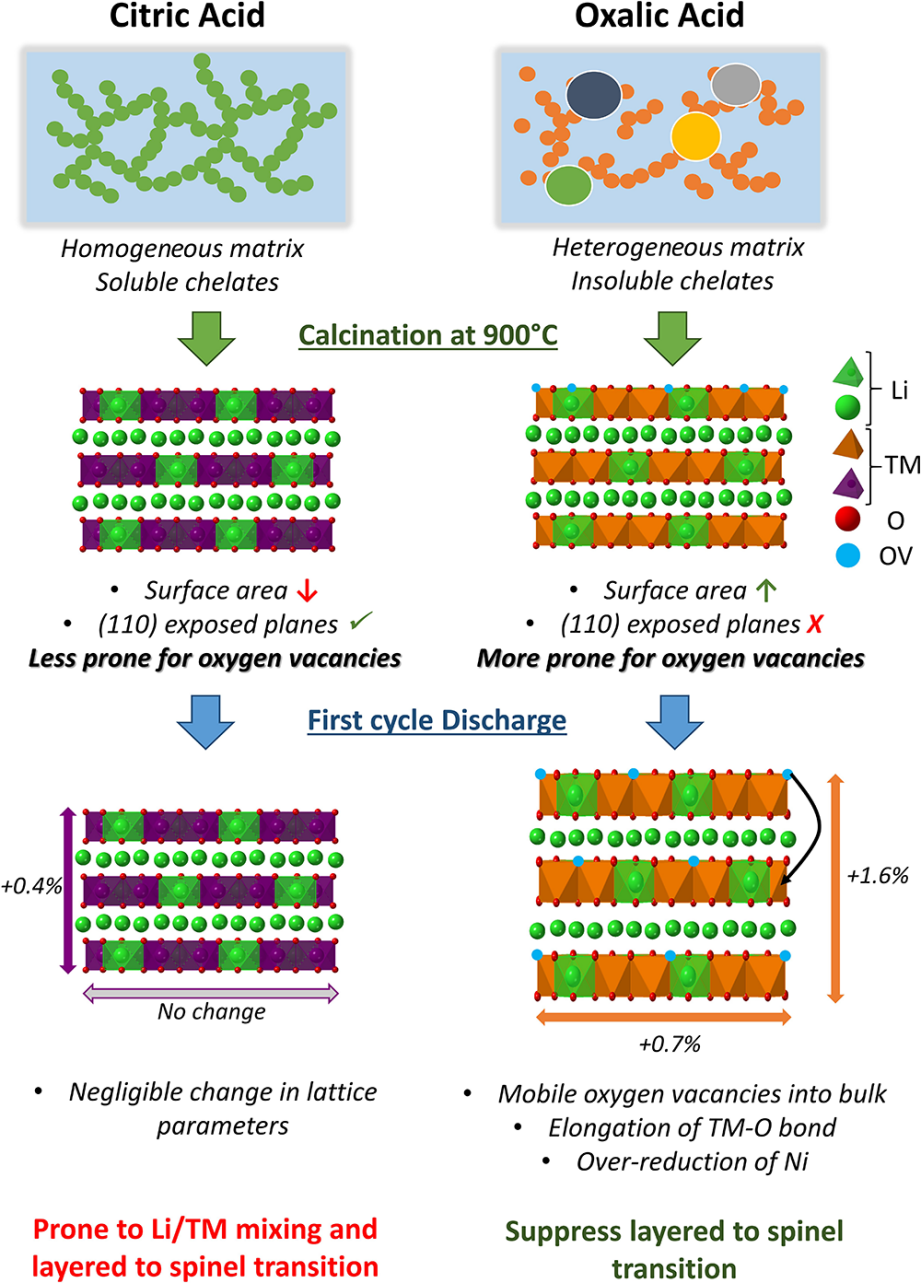
Schematic illustrating how precursor chemistry affects defect formation and electrochemical evolution in LMR oxides.

## 4 Conclusions

This study establishes that the chelating agent selection and calcination temperature play decisive roles in controlling the structural evolution, redox mechanisms, and electrochemical behavior of LMR oxides. As a case study, two chelating agents, i.e., citric and oxalic acid, and two calcination temperatures, 850 and 900 °C, were applied to synthesize cathode powders with a composition Li_1.2_Mn_0.54_Ni_0.13_Co_0.13_O_2_ via a sol–gel route. Citric acid promotes slow and homogeneous phase evolution, while oxalic acid promotes a faster, heterogeneous synthesis pathway. At 850 °C, both acid-derived samples retain high degree of stacking faults, which decrease at 900 °C due to their thermodynamic unfavorability.

In the oxalic acid-derived sample calcined at 900 °C (OA-900), the accelerated and disordered growth kinetics, along with the preferential exposure of less stable planar facets, promote the formation of oxygen vacancies, without the need for post processing. The combination of low stacking fault density, which suppresses irreversible oxygen redox by minimizing local lattice distortions, and these surface oxygen vacancies define its electrochemical response. *Operando* XRD reveals that OA-900 exhibits a marked increase in unit cell volume during the first charge–discharge cycle. This expansion is driven by the mobility of oxygen vacancies from the surface to the bulk of the structure. *Operando* and *ex situ* XANES confirm that this response, from a local structure point of view, is associated with Ni–O bond elongation due to the over-reduction of Ni during discharge. The resulting enlarged unit cell allows for cationic redox without triggering the irreversible structural collapse through transition to a spinel phase. These findings highlight how the interplay between stacking fault density and oxygen vacancy behavior, tuned through chelating agent choice and calcination temperature, can be exploited to engineer a stable, expanded lattice that limits Li/TM intermixing and preserves the layered framework during cycling.

## Supplementary Material



## Notes

Summary of Li-rich layered oxide studies highlighting chelating agents, synthesis techniques, and operando investigations; Chemical composition analysis of the synthesized LMR oxides by ICP-OES; Long-term cycling performance against LTO anodes; SEM analysis of cycled OA-900 cathode material; Calculation of *D*_Li+_ using GITT; HAADF-STEM images and EDX elemental mapping of samples at 355 °C using oxalic acid citric acid, overlay EDX elemental maps for Ni, Mn, and Co for each sample; Moduli of the Fourier transform (FT) of the *k* ^3^-weighted EXAFS signals of the three transition metals, for citric acid and oxalic acid systems at select temperatures; FAULTS refinement plots and refined parameters; HAADF-STEM and annular bright field (ABF)-STEM image; Rietveld refinement plots and refined parameters using the *R*3̅*m* space group; SEM images and corresponding histogram showing particle size distribution; EELS spectra at the O K edge, Co L_2,3_ edge, and Ni L_2,3_ edge; Changes in *d*-spacing as a function of state of charge in first charge–discharge during operando XRD; Pre-edge features at Mn K edge at selected voltage points; TM–O and TM–TM bond lengths as calculated through EXAFS fitting at different states and the EXAFS fitting parameters at Ni, Co, and Mn K-edge; XANES first derivative spectra at pristine and discharged states, at Co K-edge and Mn K edge; EELS spectra at the O K edge, Mn L_2,3_ edge, Co L_2,3_ edge, and Ni L_2,3_ edge collected of the discharged OA-900 sample (first cycle) and intensity ratio of O K edge main edge/pre-edge; XPS spectra of Ni 2p_3/2_ obtained at pristine, first cycle charge, and discharge states (PDF)
