# Supplementary material for: Impact of Chelating Agent Choice on Growth Kinetics and Defect Chemistry in Sol–Gel-Synthesized Li- and Mn-Rich Layered Cathodes
Source: ACS Appl Mater Interfaces. 2026 Mar 12;18(11):16268–81. doi: 10.1021/acsami.5c19987 (PMC13022818; doi:10.1021/acsami.5c19987)
Supplement: Supplementary file 1 [file am5c19987_si_001.pdf]

Supporting Information

Impact of Chelating Agent Choice on Growth Kinetics and Defect Chemistry in Sol–Gel Synthesized Li- and Mn-Rich Layered Cathodes

Rabail Badar Abbasi[a,b,c*], Marjan Bele[a], Giuliana Aquilanti[d], Jasper Rikkert Plaisier[d], Anton Meden[b], Luis Miguel Guerrero Mejía[a,b], Robert Dominko[a,b,c], Elena Tchernychova[a*]

[a]Department of Materials Chemistry, National Institute of Chemistry, Hajdrihova 19, Ljubljana 1000, Slovenia

[b]Faculty of Chemistry and Chemical technology, University of Ljubljana, Ljubljana 1000, Slovenia

[c]ALISTORE-European Research Institute, 80039 Amiens, France

[d]Elettra-Sincrotrone Trieste S.C.p.A., s.s. 14 km 163.5, 34149 Basovizza, Trieste, Italy

Corresponding authors: rabail.badar.abbasi@ki.si and elena.tchernychova@ki.si

Table S1. Summary of Li-rich layered oxide studies highlighting chelating agents, synthesis techniques, and *operando* investigations.

Chelating Agent used	Techniques	Link between synthesis	*Operando* study	Study of stacking faults or oxygen vacancies	Ref
**Citric acid, glycolic acid, polyvinyl pyrrolidone**	X-ray Diffraction (XRD), Rietveld Refinement, Scanning Electron Microscopy (SEM), Cyclic Voltammetry (CV), Electrochemical impedance spectroscopy (EIS), Galvanostatic charge discharge (GCD), Rate performance, Post-mortem SEM	-	-	-	1
**Oxalic acid, tartaric acid, succinic acid**	XRD, SEM, SEM-Energy-dispersive X-ray spectroscopy (EDX), GCD, Rate performance, CV, EIS	-	-	-	2
**Citric acid, ethylene diamine tetra-acetic acid (EDTA)**	XRD, Rietveld Refinement, Brunauer-Emmet Teller theory (BET), SEM-EDX, High-resolution transmission electron microscopy (HRTEM), Selected area electron diffraction (SAED), Raman Spectroscopy, GCD, dQ/dV analysis, Rate performance, EIS	EDTA synthesized LROs perform better which is attributed to lower Li^{+}/Ni^{2+} anti-site defects	-	-	3
**Glucose, citric acid, sucrose**	XRD, Rietveld Refinement, SEM, HRTEM, X-ray photoelectron spectroscopy (XPS), Laser particle analysis, GCD, dQ/dV analysis, EIS	The improved homogeneity and reduced Ni^{3+} content is attributed to the enhanced complexing behavior of sucrose.	*Ex-situ* XRD and HRTEM	-	4
**Citric acid, tartaric acid, adipic acid**	XRD, SEM, SEM-EDX, X-ray absorption near-edge spectroscopy (XANES), GCD, dQ/dV analysis	-	-	-	5
**Oxalic acid**	XRD, SEM, EDX, CV, EIS, GCD, Rate performance, dQ/dV analysis	No – highlight that energy consumption is lower when oxalic acid is used.	-	-	6

**Urea, sintered at different temperatures, using different solvents**	XRD, SEM, GCD, Rate performance, CV	800°C + water (solvent) demonstrated improved performance	-	-	7
**DL-lactic acid, effect of pH**	XRD, Rietveld Refinement, Thermogravimetric analysis (TGA), SEM, EDX, BET, GCD, Rate performance, CV, EIS	pH 5.5 and 7 improve electrochemical performance; pH 7 benefits from smaller particles.	-	-	8
**Combined use of citric acid (CA) and polymer polyacrylic acid (PAA)**	XRD, XPS, SEM, GCD, CV, EIS,	Dual chelating agents improve compositional homogeneity and reduce Li^{+}/Ni^{2+} intermixing.	-	-	9
**Oxalic acid, tartaric acid, ascorbic acid**	XRD, Rietveld Refinement, Raman Spectroscopy, SEM, HRTEM, GCD, Rate performance, CV, EIS	Ascorbic acid performs the best overall, related to small particle size and high carbon content.	-	-	10

Table S2. Chemical composition analysis of the synthesized LMR oxides by ICP-OES.

Sample	Li	Mn	Ni	Co
**Target**	1.200	0.540	0.130	0.130
**CA-850**	1.240	0.501	0.127	0.131
**CA-900**	1.224	0.512	0.129	0.133
**OA-850**	1.221	0.513	0.130	0.134
**OA-900**	1.203	0.525	0.133	0.137

Note 1. Li content

Due to the volatile nature of Li above 700°C, we added excess Li(ac) during synthesis to compensate for any Li loss. Our final oxides have a higher Li content than the target value, however structural and chemical characterization (Rietveld refinement, EELS) show that this excess Li does not create any detectible impurities or change the electronic state of the transition metals in the structure. Therefore, the redox behavior is not expected to be affected.

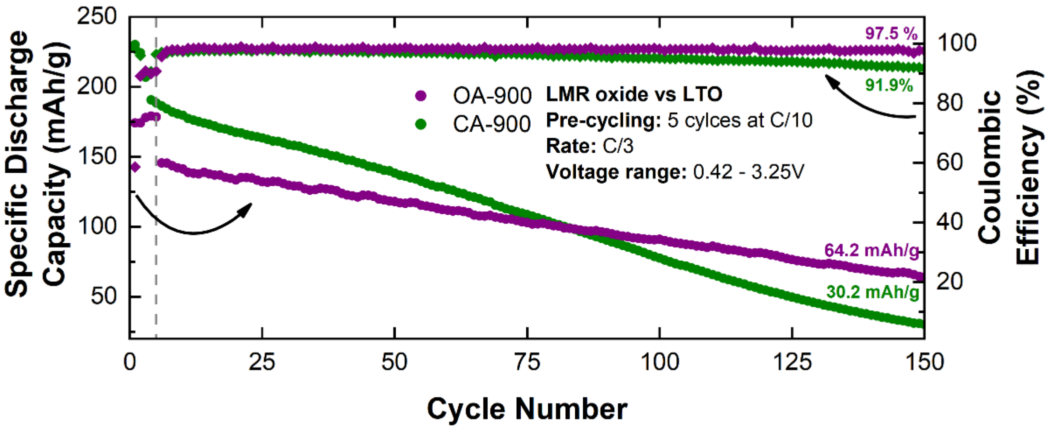

Figure S1. Long-term cycling performance and Coulombic efficiency of OA-900 and CA-900 cathodes against LTO anodes.

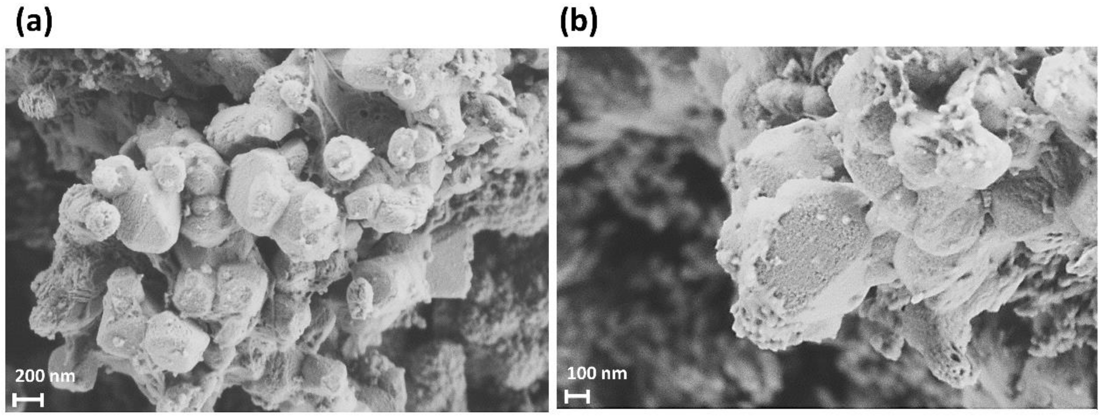

Figure S2. (a, b) SEM images of OA-900 cathode after 150 cycles against LTO anode, indicating little change in the shape of the crystals.

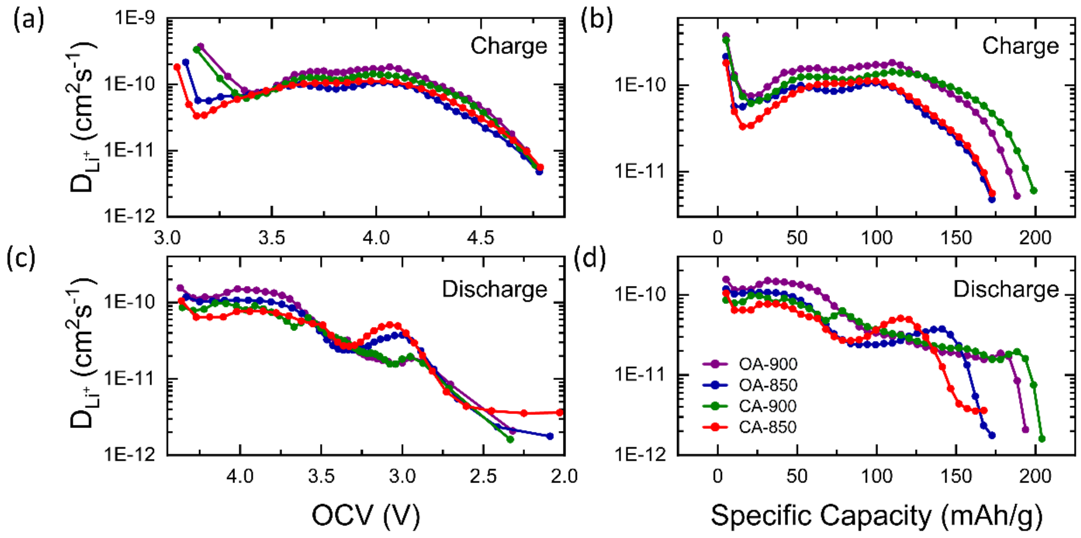

Figure S3. Lithium ion diffusion coefficients as a function of voltage during (a) charge and (c) discharge; and as a function of specific capacity during (b) charge and (d) discharge.

Note 2. Calculation of D_{Li+} using GITT

The following equation, based on Fick's laws of diffusion, is used to calculated the D_{Li+}[11]:

$$D_{Li^+} = \frac{4}{\pi}\left(\frac{m_B V_M}{M_B S}\right)^2 \left(\frac{\Delta E_s}{\tau\left(dE_\tau / d\sqrt{\tau}\right)}\right)^2 \quad (\tau \ll L^2/D_{Li^+}) \tag{S1}$$

Where:

m_B: mass of the active material in g

V_M: molar volume of active material, deduced from crystallographic data in cm^3 mol^{-1}

M_B: molecular weight of active material in g/mol

S: active geometric surface area of electrode in cm^2

ΔE_s: steady-state voltage change due to current pulse in V

τ: duration of the current pulse in s

L: thickness of electrode in cm

Considering small currents are used (C/10) for short time intervals, then E versus $\tau^{1/2}$ is assumed to be linearly related, and equation (S1) is simplified to:

$$D_{Li^+} = \frac{4}{\pi\tau}\left(\frac{m_B V_M}{M_B S}\right)^2 \left(\frac{\Delta E_s}{\Delta E_t}\right)^2 \tag{S2}$$

Where:

ΔE_t: voltage change during current pulse in V

Figure S3 shows the diffusion coefficients calculated using equation (S2) for the four cathode materials during the charge and discharge, plotted versus cell voltage and specific capacity. The use of the geometric surface area instead of the BET (Brunauer–Emmett–Teller) - derived surface area may result in discrepancies of several orders of magnitude in the calculated Li^+ diffusion coefficients. Nevertheless, as the cathodes have similar mass loadings, the D_{Li+} remains in the same order of magnitude across the four samples, allowing for reliable comparison of relative trends. During charging, the diffusion coefficient for all samples remains roughly constant at ~ 1×10^{-10} cm^2 s^{-1}; OA-900 reaches the highest value of 1.82×10^{-10} cm^2 s^{-1} at 4.0 V, then decreases for all samples at higher voltages, consistent with the sluggish kinetics of oxygen redox.[12] During discharging, the diffusion coefficients again remain near ~ 1×10^{-10} cm^2 s^{-1}, with OA-900 showing the largest value of 1.50×10^{-10} cm^2 s^{-1} at 4.0 V, before falling at lower voltages. Notably, OA-850 and CA-850 show an increase in diffusion coefficient around 3.0 V, which we attribute to possible spinel-phase formation. At both charge and discharge, the higher-temperature samples (900 °C) deliver greater capacity than the lower-temperature samples (850 °C).

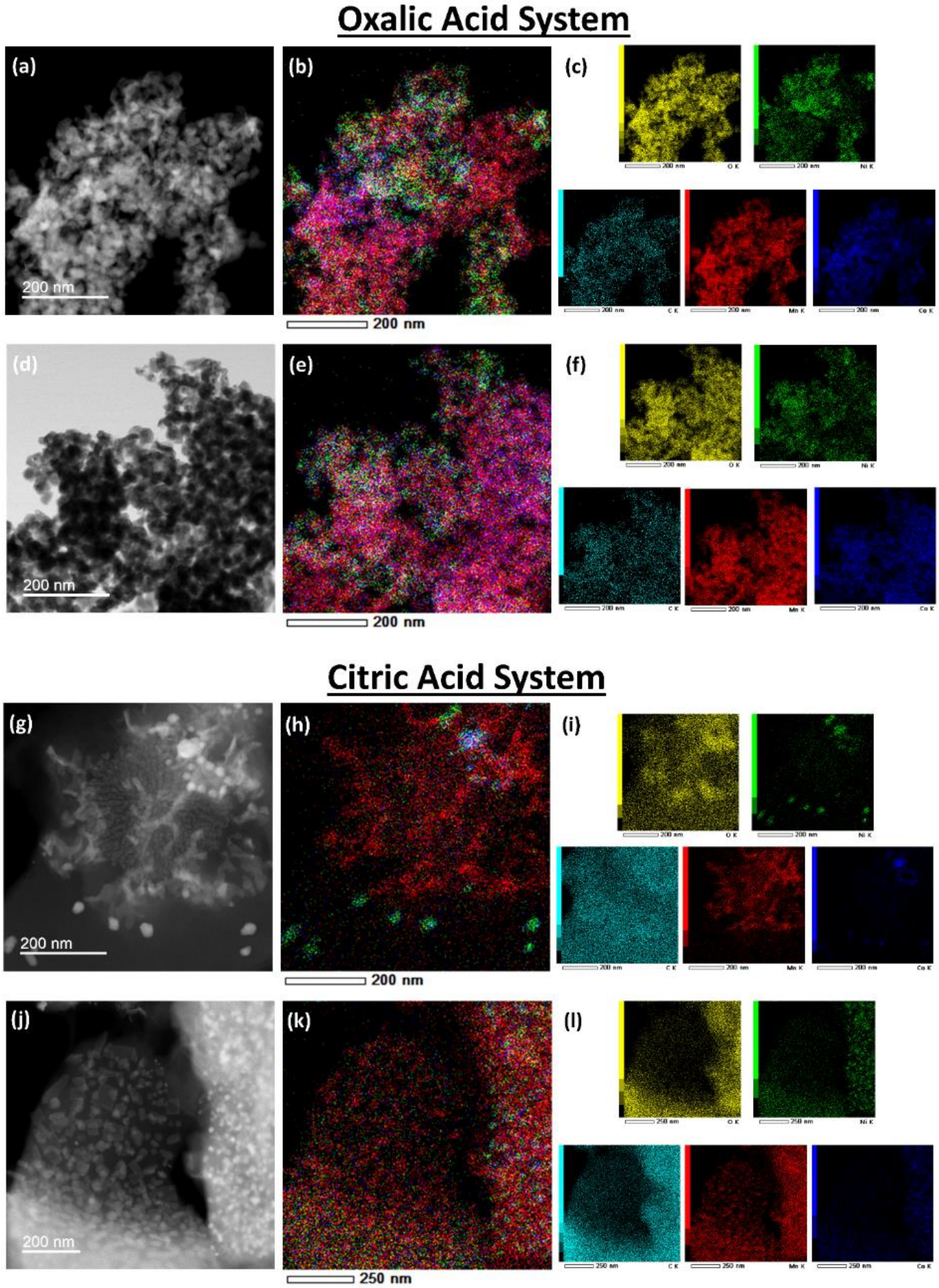

Figure S4. STEM HAADF images and EDX elemental mapping of samples at 355°C using (a-f) oxalic acid and (g-l) citric acid. (a,d,g,j) STEM-HAADF images of respective gels. (b,e,h,k) Overlay EDX elemental maps for Ni, Mn and Co. (c,f,i,l) Individual EDX elemental maps of O, Ni, C, Mn and Co for each sample.

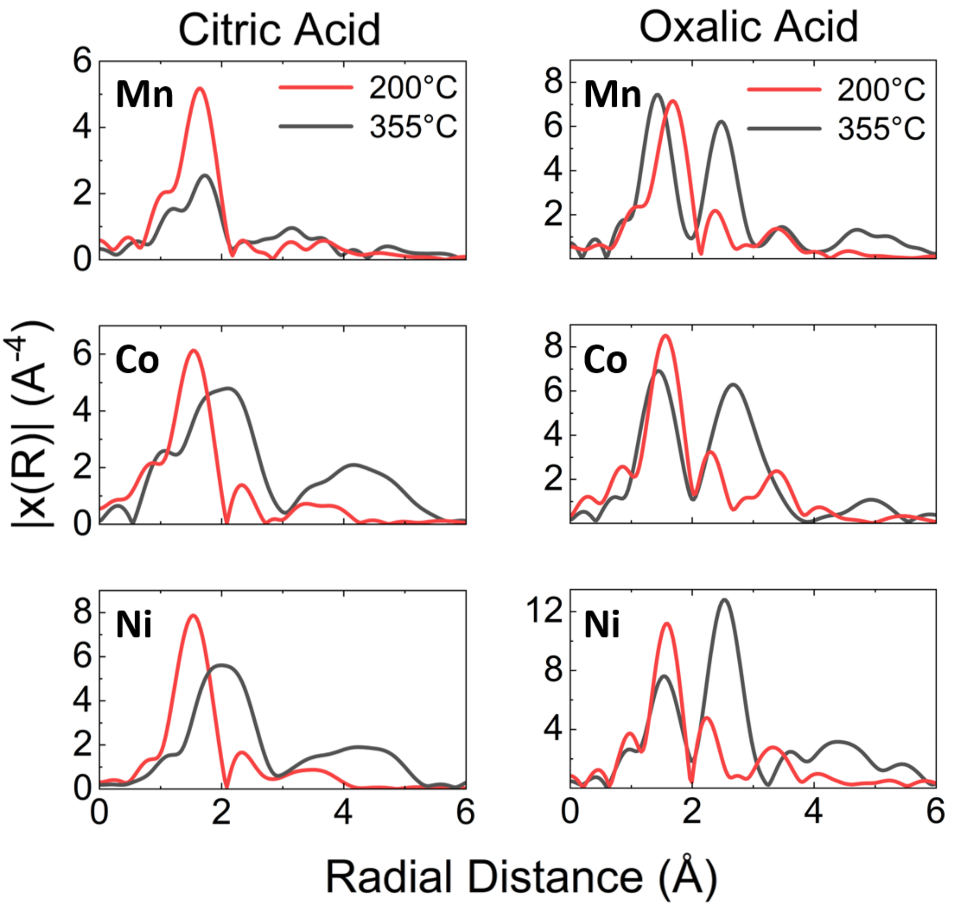

Figure S5. Moduli of the Fourier transform (FT) of the k^3-weighted EXAFS signals of the three transition metals (as labeled), for citric acid and oxalic acid systems at select temperatures.

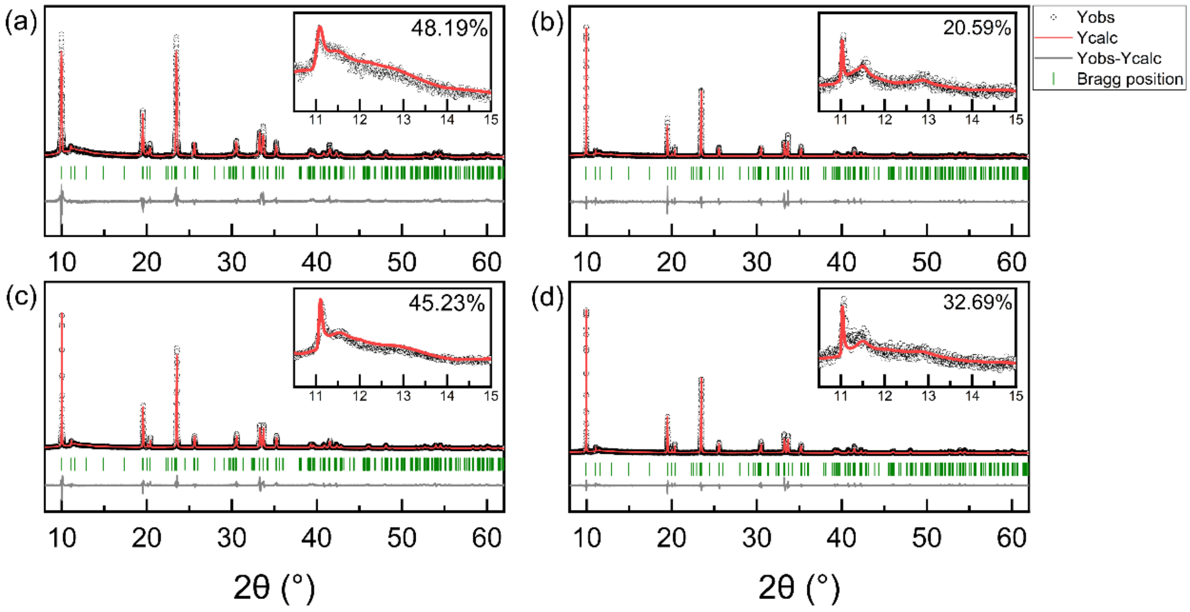

Figure S6. FAULTS refinement plots for (a) CA-850, (b) CA-900, (c) OA-850 and (d) OA-900.

Note 3. Refinement of samples with stacking faults with FAULTS[13]

Models for structural refinements are adopted from Serrano-Sevillano *et al.*, where the monoclinic Li_2MnO_3 unit cell is transformed to triclinic system with $P\bar{1}$ space group[13,14], to gain computational time. The details of the conversion of the monoclinic unit cell to the triclinic unit cell can be found in the original literature.[14] To avoid overparameterization, all TMs are approximated to Mn in the following models.

The model consists of 4 layers, where Layer 2=3=4. The atomic positions of the atoms, besides Li, were refined. The occupancy was fixed to 1 for all sites.

Table S3. Starting structural parameters used for FAULTS refinements

**Unit Cell parameters**		
**a,b (Å)**	**c (Å)**	**γ (°)**
4.9265	4.7416	60.036

**Atomic positions and occupancies**						
**Layer**	**Atom**	***x/a***	***y/b***	***z/c***	**Occupancy**	**Thermal Displacement Parameter (B_{iso})**
**1**	Li	0	0	0	1	1
	Li	1/3	1/3	0	1	1
	Li	2/3	2/3	0	1	1
**2,3 and 4**	Li	0	0	0	1	1
	Mn	0	0	0	0	1
	Mn	1/3	1/3	0	1	1
	Li	1/3	1/3	0	0	1
	Mn	2/3	2/3	0	1	1
	Li	2/3	2/3	0	0	1
	O	0.340	0.000	0.225	1	1
	O	0.650	0.000	-0.225	1	1
	O	0.000	0.340	-0.225	1	1
	O	0.340	0.650	-0.225	1	1
	O	0.650	0.340	0.225	1	1
	O	0.000	0.650	0.225	1	1

**Possible transition vectors**				
**Transition**	***x/a***	***y/b***	***z/c***	**Type of stacking**
Layer 1→Layer 2	1/3	-1/3	1/2	Rectangular
Layer 1→Layer 3	2/3	0	1/2	Parallelogram
Layer 1→Layer 4	0	-2/3	1/2	Parallelogram
Layer 2→Layer 1	1/3	-1/3	1/2	Rectangular
Layer 3→Layer 1	1/3	-1/3	1/2	Rectangular
Layer 4→Layer 1	1/3	-1/3	1/2	Rectangular

The ideal stacking (no stacking faults) consisted of Layer 1→Layer 2→Layer1 stacking. The degree of faulting was calculated from the equation below:

$$Degree\ of\ faulting\ (\%) = \frac{1-P_{L2}}{2/3} \times 100 \qquad (S3)$$

The transition probability of Layer 1→Layer 3 and Layer 1→Layer 4 are assumed to be the same.

Table S4. Refined structural parameters of CA-850 with FAULTS.

**R-Factor: 9.94870**						
**Unit Cell parameters and degree of faulting**						
**a,b (Å)**		**c (Å)**		**γ (°)**	**Degree of faulting (%)**	
4.93396(944)		4.73242(798)		60.06(45)	48.19(66)	
**Atomic positions and occupancies**						
**Layer**	**Atom**	**x/a**	**y/b**	**z/c**	**Occupancy**	**(B_{iso})**
**1**	Li	0	0	0	1	1
	Li	1/3	1/3	0	1	1
	Li	2/3	2/3	0	1	1
**2,3 and 4**	Li	0	0	0	0.6	1
	Mn	0	0	0	0.4	1
	Mn	0.33033	0.33033	0	0.9	1
	Li	0.33033	0.33033	0	0.1	1
	Mn	0.66754	0.66754	0	0.9	1
	Li	0.66754	0.66754	0	0.1	1
	O	0.32313	-0.01169	0.23246	1	1
	O	0.64531	-0.01492	-0.23609	1	1
	O	-0.01320	0.32453	-0.23288	1	1
	O	0.33149	0.64520	-0.22233	1	1
	O	0.64404	0.33253	0.22227	1	1
	O	-0.01608	0.64679	0.23543	1	1

Table S5. Refined structural parameters of CA-900 with FAULTS.

**R-Factor: 13.79481**						
**Unit Cell parameters and degree of faulting**						
**a,b (Å)**		**c (Å)**		**γ (°)**	**Degree of faulting (%)**	
4.93436(409)		4.73445(195)		60.01(7)	20.59(18)	
**Atomic positions and occupancies**						
**Layer**	**Atom**	**x/a**	**y/b**	**z/c**	**Occupancy**	**B_{iso}**
**1**	Li	0	0	0	1	1
	Li	1/3	1/3	0	1	1
	Li	2/3	2/3	0	1	1
**2,3 and 4**	Li	0	0	0	0.6	1
	Mn	0	0	0	0.4	1
	Mn	0.33236	0.33236	0	0.9	1
	Li	0.33236	0.33236	0	0.1	1
	Mn	0.66741	0.66741	0	0.9	1
	Li	0.66741	0.66741	0	0.1	1
	O	0.37839	-0.03494	0.22085	1	1
	O	0.62843	0.00581	-0.22230	1	1
	O	-0.04552	0.37027	-0.22084	1	1
	O	0.28524	0.66820	-0.21379	1	1
	O	0.69859	0.30084	0.21392	1	1
	O	0.01867	0.63111	0.22227	1	1

Table S6. Refined structural parameters of OA-850 with FAULTS.

**R-Factor: 9.13178**						
**Unit Cell parameters and degree of faulting**						
**a,b (Å)**		**c (Å)**		**γ (°)**	**Degree of faulting (%)**	
4.93573(208)		4.73494(110)		59.97(17)	45.23(9)	
**Atomic positions and occupancies**						
**Layer**	**Atom**	**x/a**	**y/b**	**z/c**	**Occupancy**	**B_{iso}**
**1**	Li	0	0	0	1	1
	Li	1/3	1/3	0	1	1
	Li	2/3	2/3	0	1	1
**2,3 and 4**	Li	0	0	0	0.6	1
	Mn	0	0	0	0.4	1
	Mn	0.33251	0.33251	0	0.9	1
	Li	0.33251	0.33251	0	0.1	1
	Mn	0.66716	0.66716	0	0.9	1
	Li	0.66716	0.66716	0	0.1	1
	O	0.30746	0.00940	0.22991	1	1
	O	0.66482	-0.03194	-0.23065	1	1
	O	0.00867	0.30795	-0.22997	1	1
	O	0.34625	0.62964	-0.22493	1	1
	O	0.62878	0.34717	0.22491	1	1
	O	-0.03258	0.66557	0.23062	1	1

Table S7. Refined structural parameters of OA-900 with FAULTS.

**R-Factor: 13.27843**						
**Unit Cell parameters and degree of faulting**						
**a,b (Å)**		**c (Å)**		**γ (°)**	**Degree of faulting (%)**	
4.93334(330)		4.73455(160)		60.03 (5)	32.69(20)	
**Atomic positions and occupancies**						
**Layer**	**Atom**	**x/a**	**y/b**	**z/c**	**Occupancy**	**B_{iso}**
**1**	Li	0	0	0	1	1
	Li	1/3	1/3	0	1	1
	Li	2/3	2/3	0	1	1
**2,3 and 4**	Li	0	0	0	0.6	1
	Mn	0	0	0	0.4	1
	Mn	0.33445	0.33445	0	0.9	1
	Li	0.33445	0.33445	0	0.1	1
	Mn	0.66854	0.66854	0	0.9	1
	Li	0.66854	0.66854	0	0.1	1
	O	0.36395	-0.04400	0.22216	1	1
	O	0.61545	0.01336	-0.22277	1	1
	O	-0.04228	0.36297	-0.22221	1	1
	O	0.31568	0.68594	-0.21685	1	1
	O	0.67666	0.31062	0.21679	1	1
	O	0.01459	0.61641	0.22272	1	1

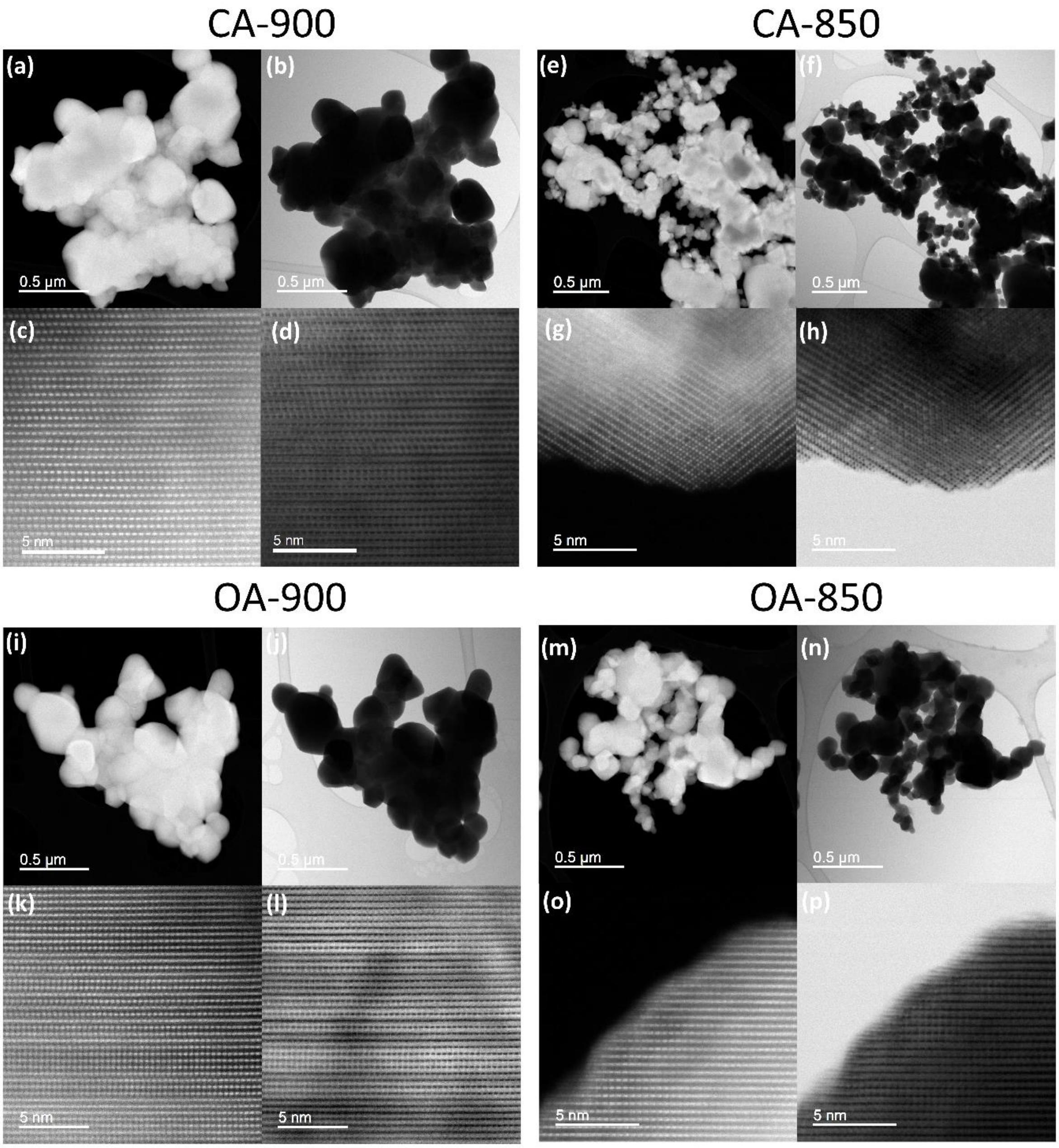

Figure S7. HAADF-STEM (a,c,e,g,i,k,m,o) and annular bright field (ABF)- STEM (b,d,f,h,j,l,n,p) images of as labeled LMR oxide samples.

Note 4. Rietveld Refinement with TOPAS[15]

The following fit parameters were refined: background coefficients, unit cell parameters, occupancies of Li and TMs and z atomic position at O-site 6c. All atoms were assumed to have the same thermal parameter (B_{iso}), which was refined. The quality of the refinement was evaluated with the R-weighted pattern (R_{wp}) value.

Due to low x-ray scattering power of O, its occupancy was not refined.

Table S8. Rietveld refinement parameters using $R\bar{3}m$ space group.

	**OA-850**	**OA-900**	**CA-850**	**CA-900**
**R_{wp}**	16.9834398	19.6252387	17.66217	19.9237059
**Lattice parameters**	a= 2.852251(45)<br>c= 14.227998(328)<br>α=γ=90°<br>β=120°	a= 2.851357(28)<br>c= 14.232455(171)<br>α=γ=90°<br>β=120°	a= 2.85101(6)<br>c= 14.20802(50)<br>α=γ=90°<br>β=120°	a= 2.851815(27)<br>c= 14.234097(181)<br>α=γ=90°<br>β=120°
**Atomic position and occupancies (Wycoff position; OF=occupancy fraction)**				
**Lithium Ion layer**	(3b) (0 0 ½)<br>OF(Li) = 0.99060(203)<br>OF(Ni) = 0.00940(203)	(3b) (0 0 ½)<br>OF(Li) = 0.99694(165)<br>OF(Ni) = 0.00306(165)	(3b) (0 0 ½)<br>OF(Li) = 0.98448(181)<br>OF(Ni) = 0.01552(181)	(3b) (0 0 ½)<br>OF(Li) = 0.99709(176)<br>OF(Ni) = 0.00291(176)
**Metal ions blend layer**	(3a) (0 0 0)<br>OF(Li)= 0.21783(351)<br>OF(Mn)= 0.55116(351)<br>OF(Ni)= 0.10610(406)<br>OF(Co)= 0.11550(351)	(3a) (0 0 0)<br>OF(Li)= 0.2322(312)<br>OF(Mn)= 0.56567(312)<br>OF(Ni)= 0.09794(353)<br>OF(Co)= 0.10100(312)	(3a) (0 0 0)<br>OF(Li)= 0.25082(469)<br>OF(Mn)= 0.58415(469)<br>OF(Ni)= 0.06699(502)<br>OF(Co)= 0.08251(469)	(3a) (0 0 0)<br>OF(Li)= 0.22837(309)<br>OF(Mn)= 0.56170(309)<br>OF(Ni)= 0.10205(356)<br>OF(Co)= 0.10496(309)
**Oxygen ions layer**	(6c) (0 0 0.25861(24))<br>OF (O) =1	(6c) (0 0 0.25822(17))<br>OF (O) =1	(6c) (0 0 0.25887(21))<br>OF (O) =1	(6c) (0 0 0.25852(20))<br>OF (O) =1
**B_{iso}**	0.94696(2544)	0.74765(2792)	0.29908(2341)	1.05861(2309)

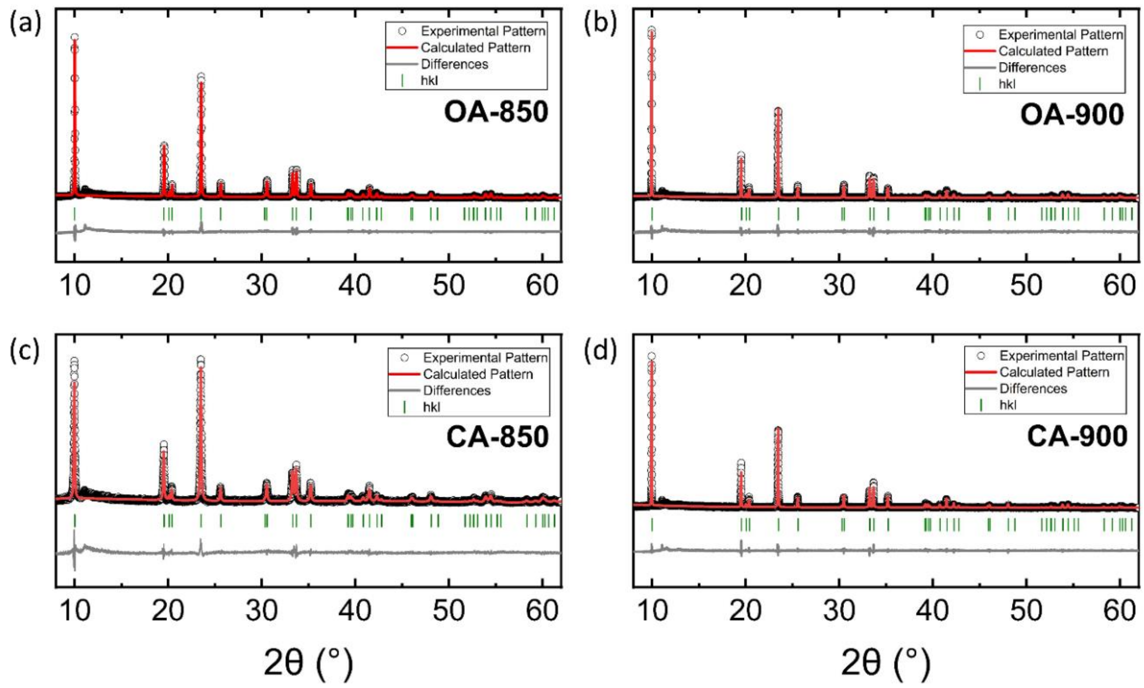

Figure S8. Rietveld refinement using $R\bar{3}m$ space group for (a) OA-850, (b) OA-900, (c) CA-850 and (d) CA-900.

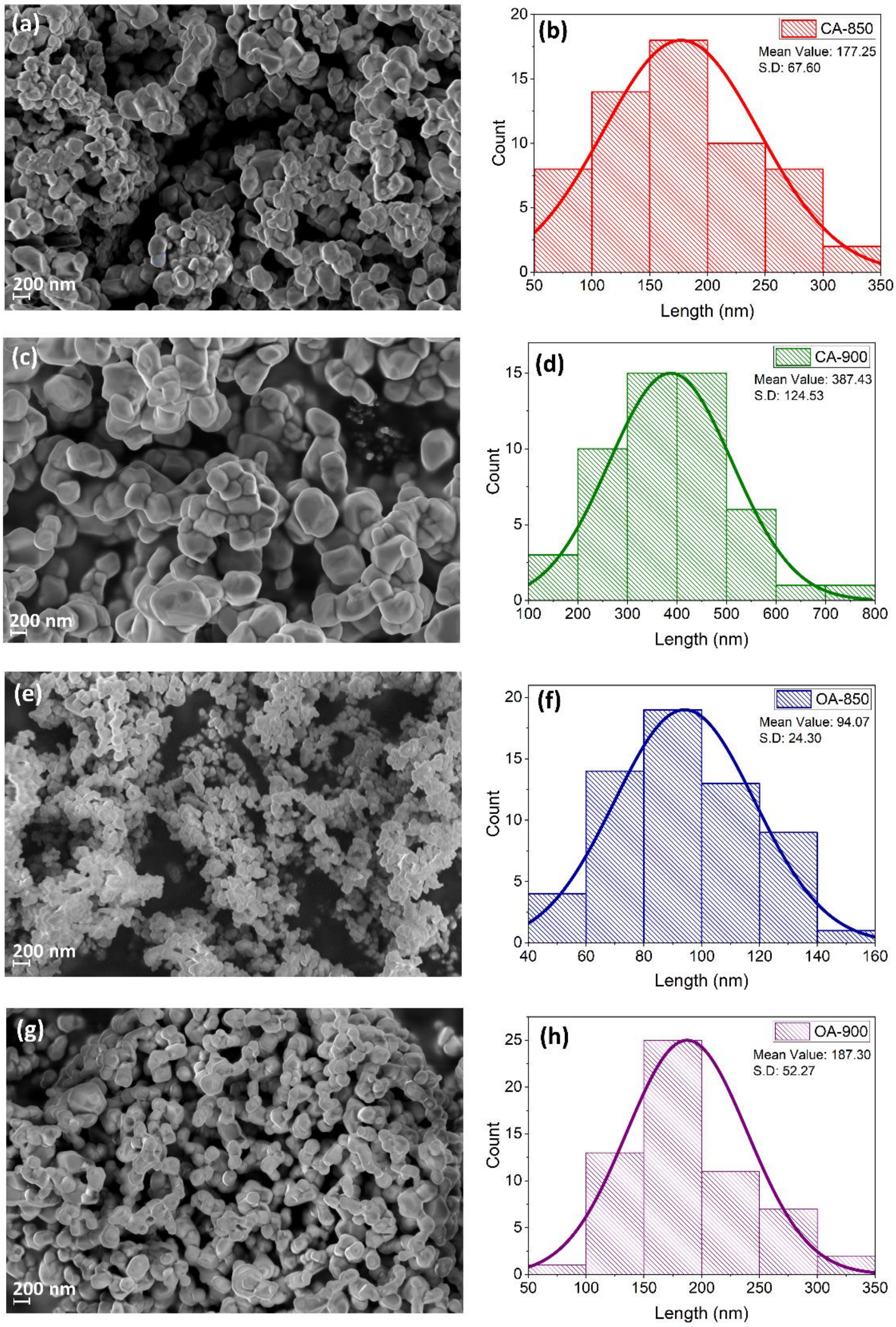

Figure S9. SEM image and corresponding histogram showing particle size distribution of (a,b) CA-850, (c,d) CA-900, (e,f) OA-850 and (g,h) OA-900.

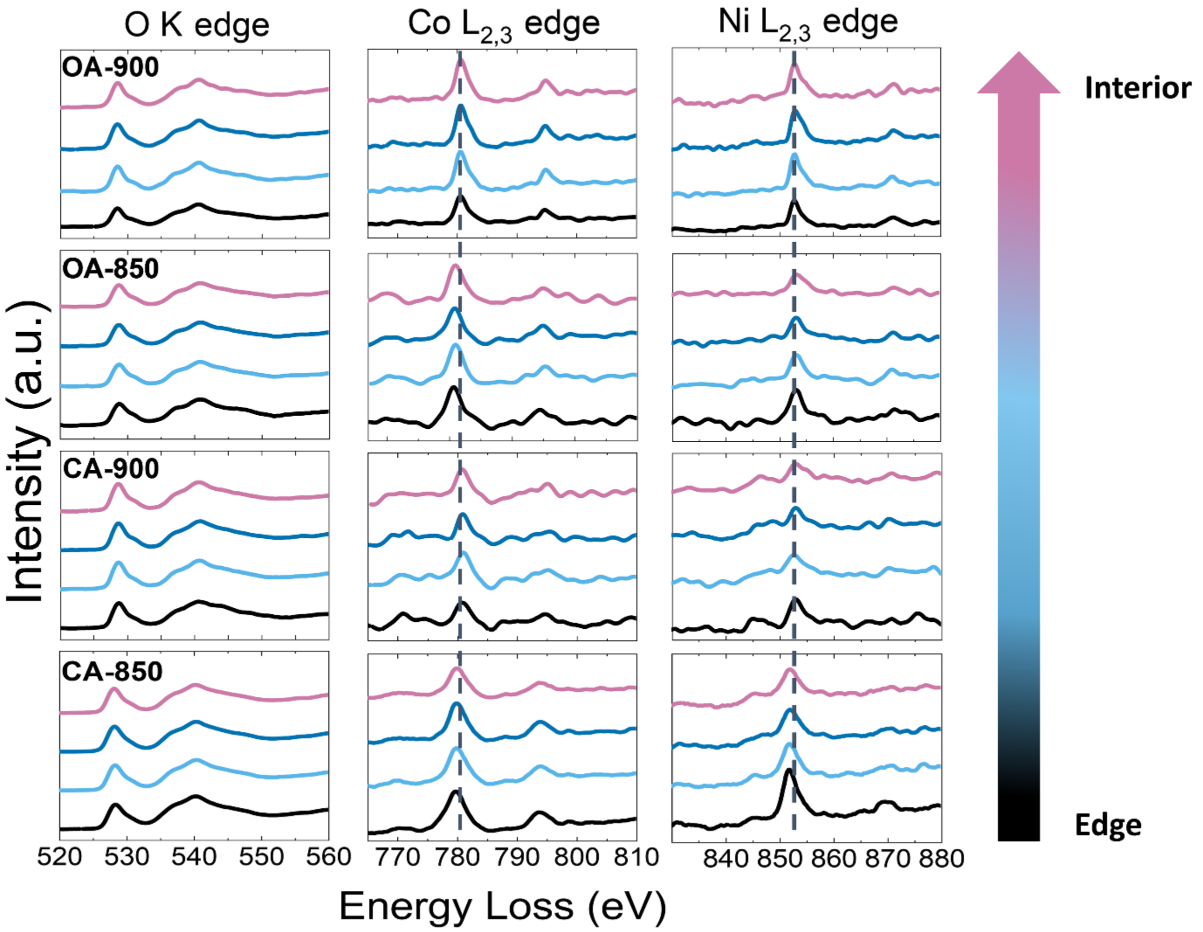

Figure S10. EELS spectra at the O K edge, Co $L_{2,3}$ edge and Ni $L_{2,3}$ edge for OA-900, OA-850, CA-900 and CA-850, as labeled. Spectras are collected at an increment of 5 nm, for a total probing depth of 20 nm.

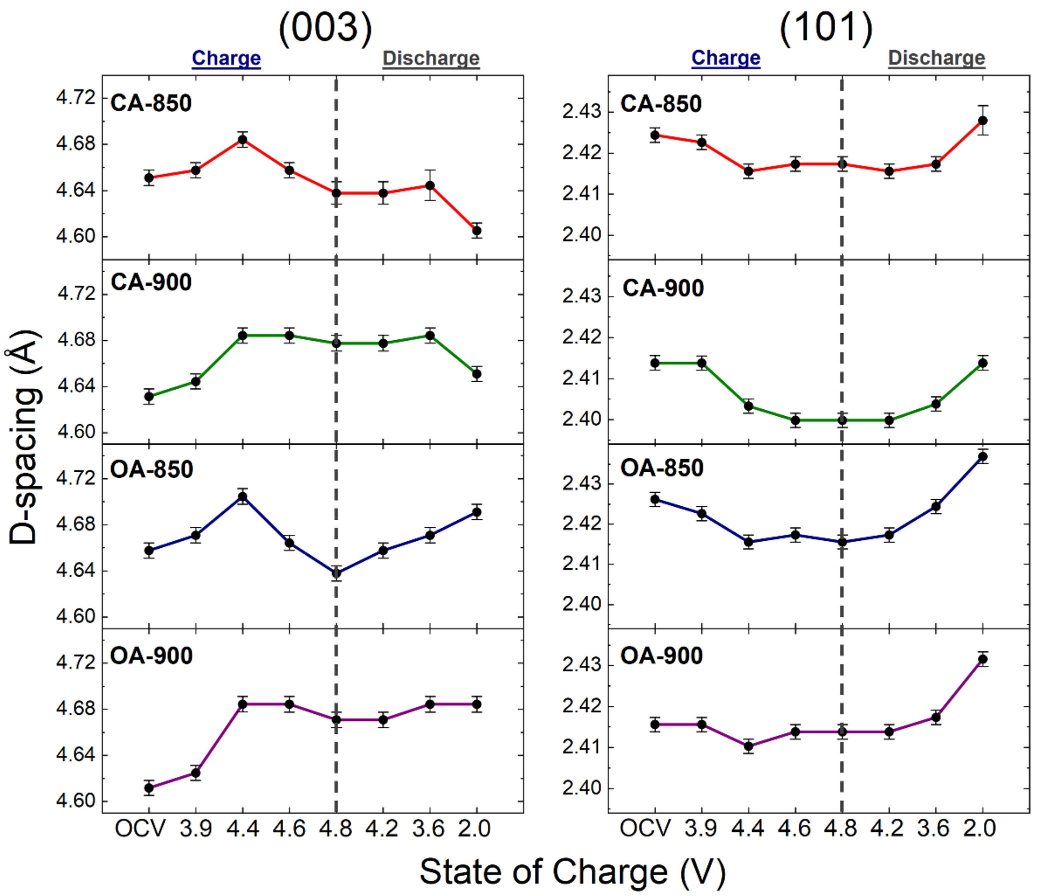

Figure S11. Changes in *d*-spacing as a function of state of charge in first charge-discharge during *operando* XRD.

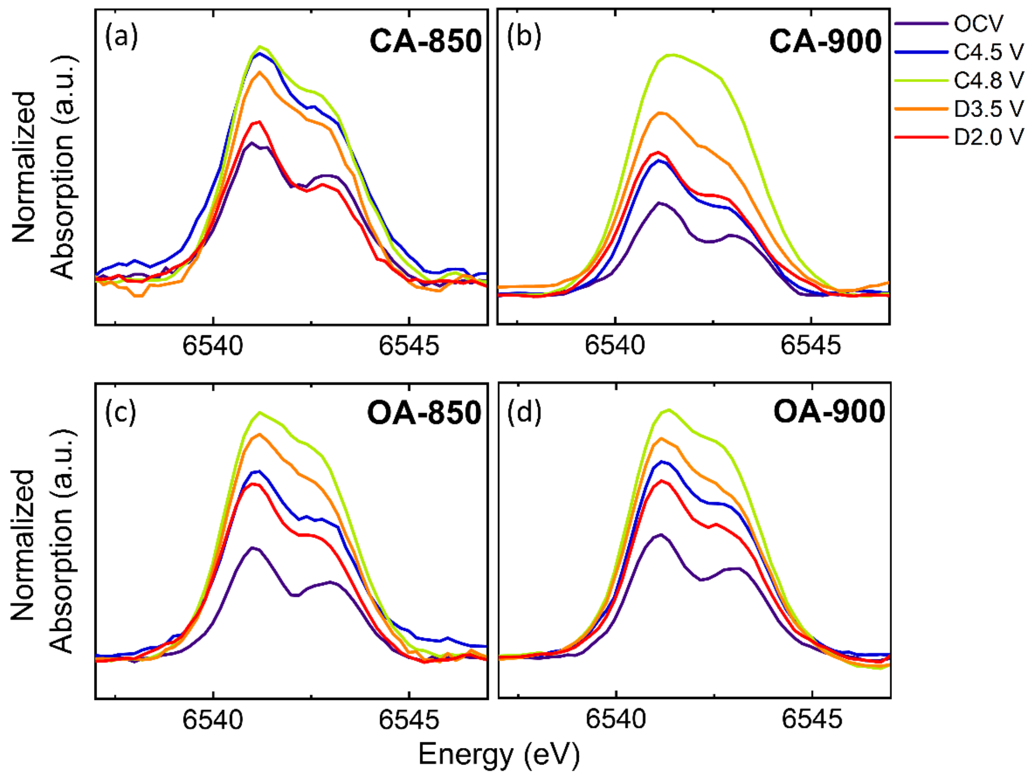

Figure S12. Pre-edge features at Mn K edge for (a) CA-850, (b) CA-900, (c) OA-850 and (d) OA-900 at selected voltage points, exhibiting the increase in intensity of the pre-edge peaks as a result of distortion in the MnO_6 octahedra.

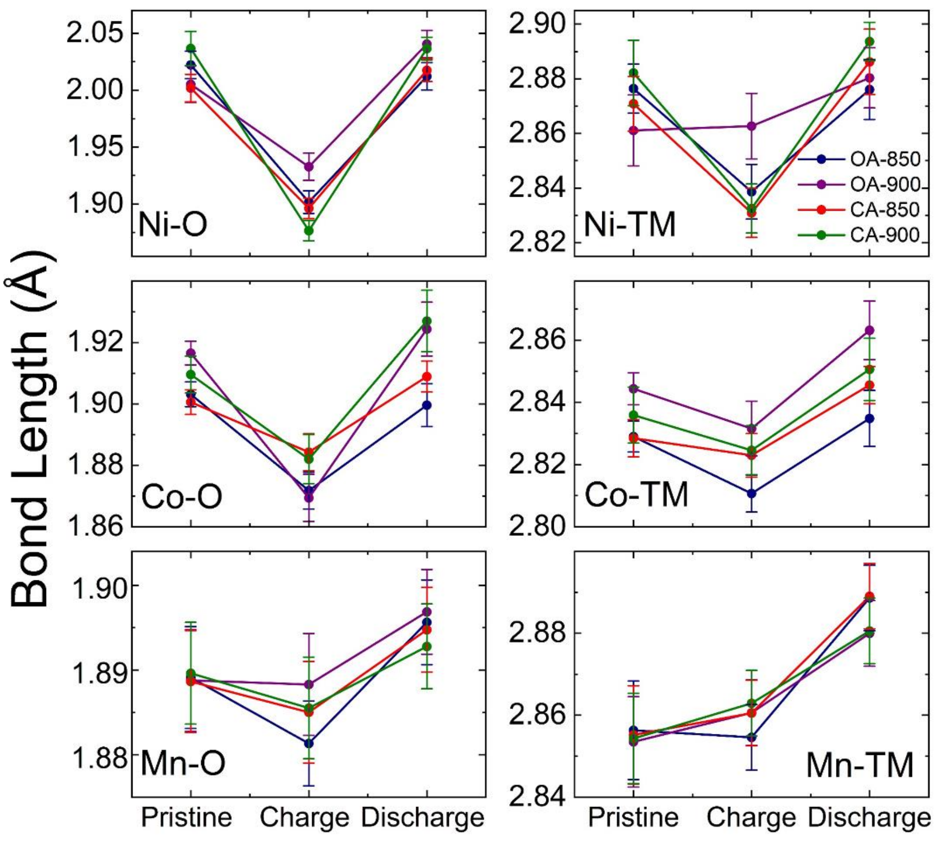

Figure S13.TM-O and TM-TM bond lengths as calculated through EXAFS fitting at different states.

Note 5. EXAFS Analysis

Quantitative analysis of Mn, Ni and Co K edge EXAFS spectra was performed with the IFEFFIT program package[16]. The structural parameters of first and second shell neighbors from the central atoms (Mn, Ni or Co) were quantitatively analyzed from EXAFS spectra by comparing the measured signals with model signals, constructed *ab inito* with the FEFF6 program code[17] from the set of scattering paths of the photoelectrons based on crystallographic data of $C2/m$ space group, to account for Li/TM ordering in the TM layer. The FEFF model comprised of O and TM, in the first and second shell, respectively. Each scattering path had the following variables: the distance of the neighboring atoms and the Debye-Waller factors (σ^2). The shell coordination numbers are kept fixed. A shift of the energy origin (ΔE_0), common to both scattering paths, was also varied. The amplitude reduction factor S_0^2 is kept fixed.

Table S9. EXAFS fitting parameters using $C2/m$ space group at Ni, Co and Mn K edge.

	**OA-900 pristine**	**OA-900 charge**	**OA-900 discharge**	**CA-900 pristine**	**CA-900 charge**	**CA-900 discharge**
**Ni-O (Å)** **σ^2 Ni-O ($Å^2$)**	2.005 (16)[a] 0.008 (3)	1.933 (12) 0.004 (2)	2.040(12) 0.013(2)	2.037(15) 0.009(3)	1.876(9) 0.007(1)	2.037 (10) 0.012 (2)
**Ni-TM (Å)** **σ^2 Ni-TM ($Å^2$)**	2.861 (13)[a] 0.004 (2)	2.863 (12) 0.003 (2)	2.880 (11) 0.007(1)	2.882(12) 0.004(2)	2.832(9) 0.005(1)	2.893 (7) 0.005 (1)
**E_0 Ni (eV)**	8342.5(8)	8345.8(7)	8344.3(6)	8344.7(7)	8340.9(6)	8345.5(5)
**R factor**	0.032	0.027	0.020	0.030	0.017	0.013
**Co-O (Å)** **σ^2 Co-O ($Å^2$)**	1.916(4)[a] 0.002(1)	1.869(7) 0.003(1)	1.924(8) 0.003(1)	1.900(5) 0.002(1)	1.882(8) 0.007(1)	1.927(10) 0.008(2)
**Co-TM (Å)** **σ^2 Co-O ($Å^2$)**	2.844(5)[b] 0.001(1)	2.831(8) 0.001(1)	2.863(9) 0.0002(1)	2.825(6) 0.003(1)	2.825(8) 0.003(1)	2.850(10) 0.002(1)
**E_0 Co (eV)**	7721.0(3)	7718.8(6)	7722.0(5)	7719.8(5)	7719.3(6)	7721.7(6)
**R factor**	0.004	0.015	0.016	0.011	0.017	0.031
**Mn-O (Å)** **σ^2 Mn-O ($Å^2$)**	1.888(6)[a] 0.001(1)	1.888(6) 0.004(1)	1.897(5) 0.003(1)	1.889(6) 0.001(1)	1.885(6) 0.004(1)	1.893(5) 0.004(1)
**Mn-TM (Å)** **σ^2 Mn-TM ($Å^2$)**	2.853(11)[b] 0.004(1)	2.860(8) 0.004(1)	2.880(8) 0.004(1)	2.854(11) 0.004(1)	2.863(8) 0.003(1)	2.880(8) 0.003(1)

**E_0 Mn (eV)**	6550.7(6)	6551.2(5)	6551.7(5)	6550.6(6)	6550.8(5)	6550.9(4)
**R factor**	0.022	0.017	0.015	0.023	0.016	0.014

	**CA-850 pristine**	**CA-850 charge**	**CA-850 discharge**	**OA-850 pristine**	**OA-850 charge**	**OA-850 discharge**
**Ni-O (Å)<br>σ^2 Ni-O (Å^2)**	2.000(12)[a]<br>0.006(2)	1.899(7)<br>0.002(1)	2.015 (10)<br>0.002(1)	2.022(12)<br>0.009(2)	1.902(10)<br>0.002(2)	2.012(13)<br>0.005(2)
**Ni-TM (Å)<br>σ^2 Ni-TM (Å^2)**	2.875(10)[a]<br>0.004(1)	2.839(8)<br>0.004(1)	2.891(10)<br>0.004 (1)	2.876(10)<br>0.005(1)	2.838(10)<br>0.004(1)	2.876(12)<br>0.005(2)
**E_0 Ni (eV)**	8343.4(7)	8342.1(6)	8343.2(6)	8244.4(6)	8343.0(7)	8342.5(7)
**R factor**	0.026	0.014	0.022	0.024	0.026	0.033
**Co-O (Å)<br>σ^2 Co-O (Å^2)**	1.900(5)[a]<br>0.002(1)	1.884(6)<br>0.008(1)	1.908(5)<br>0.005(1)	1.903(4)<br>0.002(1)	1.873(6)<br>0.003(1)	1.899(8)<br>0.005(1)
**Co-TM (Å)<br>σ^2 Co-O (Å^2)**	2.830(6)[b]<br>0.001(1)	2.823(7)<br>0.005(1)	2.845(6)<br>0.002(1)	2.829(5)<br>0.002(1)	2.811(7)<br>0.002(1)	2.835(9)<br>0.004(1)
**E_0 Co (eV)**	7718.8(4)	7718.4(5)	7718.0(4)	7719.0(3)	7717.7(5)	7716.7(6)
**R factor**	0.007	0.013	0.007	0.006	0.010	0.017
**Mn-O (Å)<br>σ^2 Mn-O (Å^2)**	1.888(6)[a]<br>0.002(1)	1.885(6)<br>0.004(1)	1.895(5)<br>0.003(1)	1.889(6)<br>0.002(1)	1.881(6)<br>0.004(1)	1.896(6)<br>0.004(1)
**Mn-TM (Å)<br>σ^2 Mn-TM (Å^2)**	2.855(12)[b]<br>0.005(2)	2.861(8)<br>0.004(1)	2.890(8)<br>0.004(1)	2.856(12)<br>0.005(2)	2.855(8)<br>0.004(1)	2.888(8)<br>0.004(1)
**E_0 Mn (eV)**	6550.4(6)	6550.6(5)	6550.5(4)	6550.6(6)	6550.2(5)	6550.5(4)
**R factor**	0.023	0.017	0.012	0.023	0.016	0.014

[a] CN=6, [b] CN=4

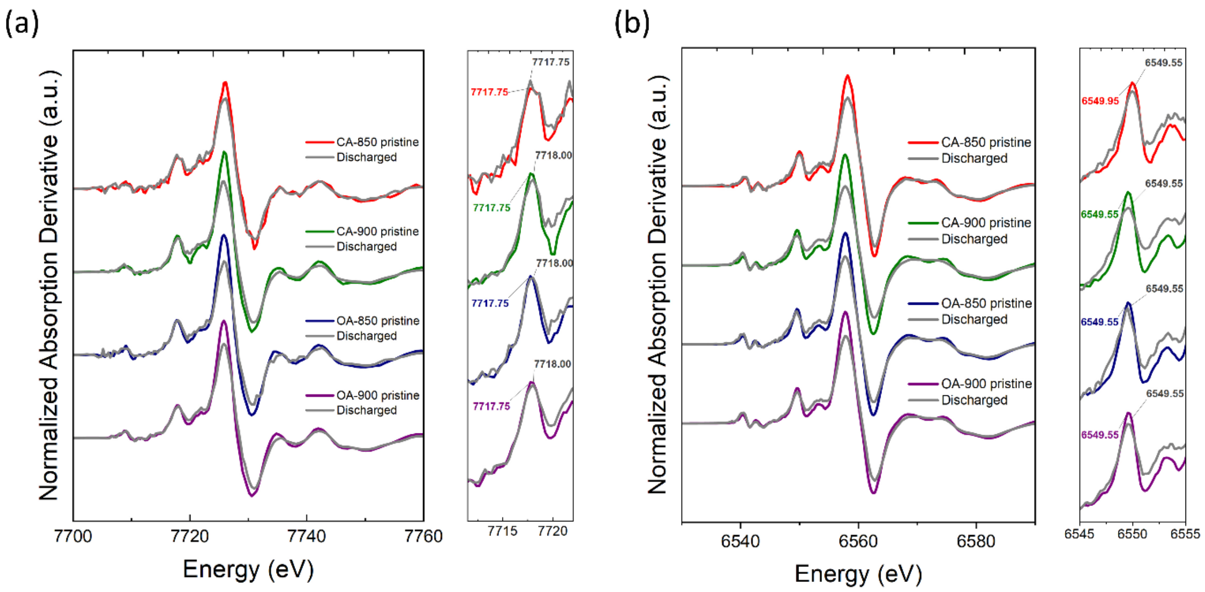

Figure S14. XANES normalized first derivative spectra of LMR oxide samples at pristine and discharged states, at (a) Co K edge and (b) Mn K edge, with zoomed-in inset of first peak in relation to oxidation state.

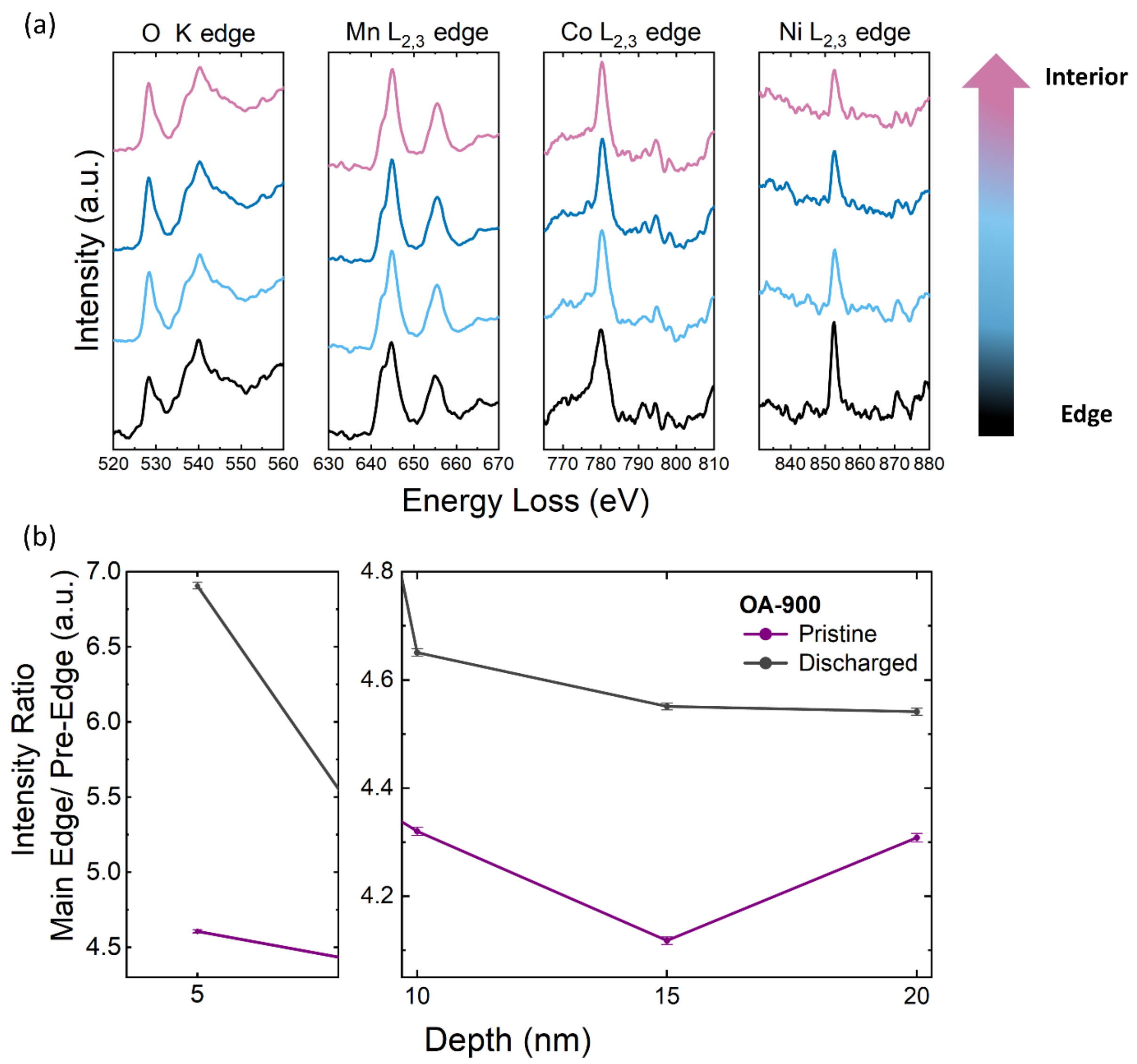

Figure S15. (a) STEM-EELS spectra at the O K edge, Mn $L_{2,3}$ edge, Co $L_{2,3}$ edge and Ni $L_{2,3}$ edge collected of discharged OA-900 sample (first cycle). Spectras are collected at an increment of 5 nm, for a total probing depth of 20 nm, (b) Intensity ratio of O K edge main edge/pre-edge as a function of depth for OA-900 at pristine and discharged state.

Note 6. STEM-EELS O K edge analysis

The intensity ratio of the O K edge main peak to the pre-peak was calculated by integrating the peak intensity at the full width at half maximum for each peak. The energy window for integration was maintained at 2 eV and 9 eV for the pre-peak and main peak, respectively.

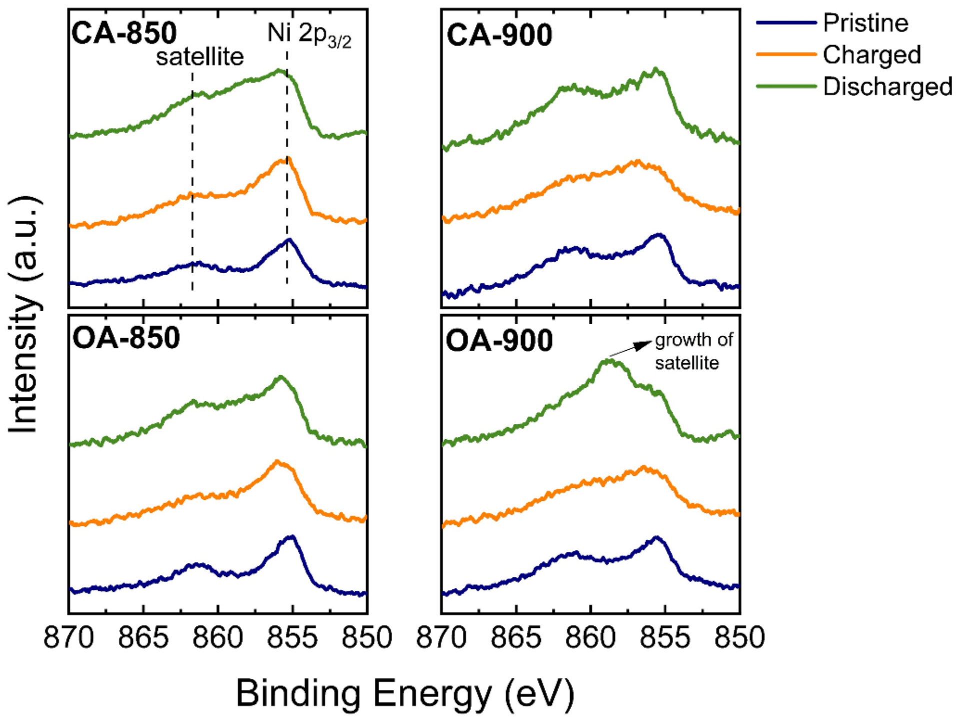

Figure S16. XPS spectra of Ni $2p_{3/2}$ obtained at pristine, 1st cycle charge and discharge states of the as-labeled cathodes.

References

(1) Wu, Q.; Zhao, L.; Wu, J. Effects of Chelating Agents on the Performance of $Li_{1.2}Mn_{0.54}Ni_{0.13}Co_{0.13}O_2$ as Cathode Material for Li-Ion Battery Prepared by Sol–Gel Method. *J. Sol-Gel Sci. Technol.* **2017**, *82* (2), 335–343 10.1007/s10971-017-4338-7.

(2) Zhao, T.; Chen, S.; Li, L.; Zhang, X.; Chen, R.; Belharouak, I.; Wu, F.; Amine, K. Synthesis, Characterization, and Electrochemistry of Cathode Material $Li[Li_{0.2}Co_{0.13}Ni_{0.13}Mn_{0.54}]O_2$ Using Organic Chelating Agents for Lithium-Ion Batteries. *J. Power Sources* **2013**, *228*, 206–213. 10.1016/j.jpowsour.2012.11.099.

(3) Abdel-Ghany, A. E.; Hashem, A. M.; Mauger, A.; Julien, C. M. Effects of Chelators on the Structure and Electrochemical Properties of Li-Rich $Li_{1.2}Mn_{0.54}Ni_{0.13}Co_{0.13}O_2$ Cathode Materials. *J. of Solid State Electrochem.* **2020**, *24* (11), 3157–3172 10.1007/s10008-020-04789-7.

(4) Li, L.; Xu, M.; Chen, Z.; Zhou, X.; Zhang, Q.; Zhu, H.; Wu, C.; Zhang, K. High-Performance Lithium-Rich Layered Oxide Materials: Effects of Chelating Agents on Microstructure and Electrochemical Properties. *Electrochim. Acta* **2015**, *174*, 446–455 10.1016/j.electacta.2015.05.171.

(5) Zhao, T.; Chen, S.; Li, L.; Zhang, X.; Wu, H.; Wu, T.; Sun, C.-J.; Chen, R.; Wu, F.; Lu, J.; Amine, K. Organic-Acid-Assisted Fabrication of Low-Cost Li-Rich Cathode Material ($Li[Li_{1/6}Fe_{1/6}Ni_{1/6}Mn_{1/2}]O_2$) for Lithium–Ion Battery. *ACS Appl. Mater. Interfaces* **2014**, *6* (24), 22305–22315 10.1021/am5062882.

(6) Jin, X.; Xu, Q.; Yuan, X.; Zhou, L.; Xia, Y. Synthesis, Characterization and Electrochemical Performance of $Li[Li_{0.2}Mn_{0.54}Ni_{0.13}Co_{0.13}]O_2$ cathode Materials for Lithium-Ion Batteries. *Electrochim. Acta* **2013**, *114*, 605–610 10.1016/j.electacta.2013.10.091.

(7) He, D.; Guo, Q.; Yin, H.; Li, J.; Gong, Z. Sol-Gel Combustion Synthesis of $Li_{1.2}Mn_{0.54}Ni_{0.13}Co_{0.13}O_2$ as Cathode Materials for Lithium Ion Batteries. *Int. J. Electrochem. Sci.* **2017**, *12* (1), 455–465 10.20964/2017.01.18.

(8) Chen, C.; Wu, H.; Zhou, D.; Xu, D.; Zhou, Y.; Guo, J. Sol-Gel Synthesis of Nano $Li_{1.2}Mn_{0.54}Ni_{0.13}Co_{0.13}O_2$ Cathode Materials Using DL-Lactic Acid as Chelating Agent. *Ceram. Int.* **2021**, *47* (5), 6270–6278 10.1016/j.ceramint.2020.10.205.

(9) Liao, D.; Xia, C.; Xi, X.; Zhou, C.; Xiao, K.; Chen, X.; Qin, S. Sol–Gel Preparation of Li-Rich Layered Cathode Material for Lithium Ion Battery with Polymer Polyacrylic Acid + Citric Acid Chelators. *J. Solgel Sci. Technol.* **2016**, *78* (2), 403–410 10.1007/s10971-016-3956-9.

(10) Prettencia, L. J.; Soundarrajan, E.; Roselin Ranjitha, M.; Kalaivani, R.; Raghu, S. Sol-Gel Route Synthesis of High Energy Density Li $[Li_{0.2}Ni_{0.3}Mn_{0.7}]$ O_2 Cathode with Controlled Structure, Morphology and Enhanced Electrochemical Performance. *Energy Storage* **2023**, *5* (4), e427 10.1002/est2.427.

(11) Kim, J.; Park, S.; Hwang, S.; Yoon, W.-S. Principles and Applications of Galvanostatic Intermittent Titration Technique for Lithium-Ion Batteries. *J. Electrochem. Sci. Technol* **2021**, *13* (1), 19–31 10.33961/jecst.2021.00836.

(12) Zheng, J.; Shi, W.; Gu, M.; Xiao, J.; Zuo, P.; Wang, C.; Zhang, J.-G. Electrochemical Kinetics and Performance of Layered Composite Cathode Material $Li[Li_{0.2}Ni_{0.2}Mn_{0.6}]O_2$. *J. Electrochem. Soc.* **2013**, *160* (11), A2212 10.1149/2.090311jes.

(13) Casas-Cabanas, M.; Reynaud, M.; Rikarte, J.; Horbach, P.; Rodriguez-Carvajal, J. FAULTS: A Program for Refinement of Structures with Extended Defects. *J. Appl. Cryst* **2016**, *49* (6), 2259–2269 10.1107/S1600576716014473.

(14) Serrano-Sevillano, J.; Reynaud, M.; Saracibar, A.; Altantzis, T.; Bals, S.; van Tendeloo, G.; Casas-Cabanas, M. Enhanced Electrochemical Performance of Li-Rich Cathode Materials through Microstructural Control. *Phys. Chem. Chem. Phys* **2018**, *20* (35), 23112–23122 10.1039/C8CP04181D.

(15) Dinnebier, R. E.; Leineweber, A.; Evans, J. S. O. *Practical Powder Diffraction Pattern Analysis Using TOPAS*; De Gruyter: Berlin, Boston, 2019 10.1515/9783110461381.

(16) Ravel, B.; Newville, M. ATHENA, ARTEMIS, HEPHAESTUS: Data Analysis for X-Ray Absorption Spectroscopy Using IFEFFIT. *J. Synchrotron. Rad* **2005**, *12* (4), 537–541 10.1107/S0909049505012719.

(17) J.J. Rehr; R.C. Albers; S.L. Zabinsky. High-Order Multiple-Scattering Calculations of x-Ray-Absorption Fine Structure. *Phys. Rev. Lett* **1992**, *69* (23), 3397–3400 10.1103/PhysRevLett.69.3397.
